# Role of autophagy-related proteins ATG8f and ATG8h in the maintenance of autophagic activity in *Arabidopsis* roots under phosphate starvation

**DOI:** 10.3389/fpls.2023.1018984

**Published:** 2023-06-26

**Authors:** Li-Yen Lin, Hong-Xuan Chow, Chih-Hao Chen, Nobutaka Mitsuda, Wen-Chun Chou, Tzu-Yin Liu

**Affiliations:** ^1^ Institute of Bioinformatics and Structural Biology, College of Life Sciences and Medicine, National Tsing Hua University, Hsinchu, Taiwan; ^2^ Bioproduction Research Institute, National Institute of Advanced Industrial Science and Technology (AIST), Tsukuba, Japan; ^3^ Department of Life Science, College of Life Sciences and Medicine, National Tsing Hua University, Hsinchu, Taiwan

**Keywords:** *Arabidopsis*, phosphate starvation, autophagy, autophagy-related protein 8 (ATG8), lateral root

## Abstract

Nutrient starvation-induced autophagy is a conserved process in eukaryotes. Plants defective in autophagy show hypersensitivity to carbon and nitrogen limitation. However, the role of autophagy in plant phosphate (Pi) starvation response is relatively less explored. Among the core autophagy-related (*ATG*) genes, *ATG8* encodes a ubiquitin-like protein involved in autophagosome formation and selective cargo recruitment. The *Arabidopsis thaliana ATG8* genes, *AtATG8f* and *AtATG8h*, are notably induced in roots under low Pi. In this study, we show that such upregulation correlates with their promoter activities and can be suppressed in the *phosphate response 1* (*phr1*) mutant. Yeast one-hybrid analysis failed to attest the binding of the *At*PHR1 transcription factor to the promoter regions of *AtATG8f* and *AtATG8h*. Dual luciferase reporter assays in *Arabidopsis* mesophyll protoplasts also indicated that *At*PHR1 could not transactivate the expression of both genes. Loss of *At*ATG8f and *At*ATG8h leads to decreased root microsomal-enriched ATG8 but increased ATG8 lipidation. Moreover*, atg8f/atg8h* mutants exhibit reduced autophagic flux estimated by the vacuolar degradation of ATG8 in the Pi-limited root but maintain normal cellular Pi homeostasis with reduced number of lateral roots. While the expression patterns of *AtATG8f* and *AtATG8h* overlap in the root stele, *AtATG8f* is more strongly expressed in the root apex and root hair and remarkably at sites where lateral root primordia develop. We hypothesize that Pi starvation-induction of *At*ATG8f and *At*ATG8h may not directly contribute to Pi recycling but rely on a second wave of transcriptional activation triggered by PHR1 that fine-tunes cell type-specific autophagic activity.

## Introduction

Autophagy is a highly conserved catabolic process in eukaryotes that maintains cellular homeostasis and contributes to stress adaptation (Marshall and Vierstra, 2018; Gross and Graef, 2020). It begins with the induction and nucleation of isolation membranes, followed by the formation of cup-shaped pre-autophagosome structures called phagophores, which eventually mature into closed double-membrane autophagosomes (Yoshimoto and Ohsumi, 2018; Wun et al., 2020). During the process, damaged or dispensable cytoplasmic components, protein aggregates, and dysfunctional organelles are enclosed in the autophagosome (Yoshimoto and Ohsumi, 2018; Wun et al., 2020). As the autophagosome reaches the vacuole or the lysosome, its outer membrane fuses with the vacuolar/lysosomal membrane and releases the autophagic bodies for degradation (Yoshimoto and Ohsumi, 2018; Wun et al., 2020). The breakdown products are then recycled for energy production or usage in biosynthetic pathways (Yoshimoto and Ohsumi, 2018; Wun et al., 2020). The biogenesis of autophagosome is stepwise and dynamic, and is driven by a large number of autophagy-related (ATG) genes that can be categorized into four functional groups (Yoshimoto and Ohsumi, 2018; Wun et al., 2020). The ATG1/ATG13 kinase complex stimulates autophagosome formation in response to the phosphorylation status of ATG13 (Kamada et al., 2000; Suttangkakul et al., 2011). The class III phosphatidylinositol 3-kinase (PI3K) complex containing VACUOLAR PROTEIN SORTING 34 (VPS34), ATG6 and ATG14, incorporates the phosphatidylinositol 3-phosphate (PI3P) phospholipids into the expanding phagophore (Russell et al., 2013). The ATG2-ATG18-ATG9 complex localizes to the edge of phagophore and delivers the lipid molecules for its expansion (Mari and Reggiori, 2007; Zhuang et al., 2017). The ATG12 and ATG8 ubiquitin-like conjugation systems, which consist of the E1-like ATG7, the E2-like ATG3 and ATG10 and the E3-like ATG12-ATG5 conjugate together with ATG16, participate in autophagosome maturation (Geng and Klionsky, 2008). Of note, the ubiquitin-like protein ATG8, through its covalent conjugation to the lipid phosphatidylethanolamine (​PE), plays a central role in both bulk and selective autophagy (Marshall and Vierstra, 2018; Bu et al., 2020). Although ATG8 interacts with diverse receptors or adaptor proteins to recruit specific cargos for degradation, autophagy-independent function of ATG8 has also been reported (Marshall and Vierstra, 2018; Bu et al., 2020). In addition, ATG8 is used as a reliable marker to monitor autophagic degradation activity upon the inhibition of vacuolar/lysosomal degradation by protease inhibitors (Klionsky et al., 2021).

Unlike a single-copy *ATG8* gene in yeast and algae, the plant *ATG8* gene family has significantly expanded and some members are upregulated under various biotic and abiotic stresses (Kellner et al., 2017; Bu et al., 2020; Qi et al., 2021). Selective interaction of various ATG8 isoforms (ATG8s) with their protein targets may contribute to the diversification of autophagy pathways in plants (Svenning et al., 2011; Kellner et al., 2017; Boycheva Woltering and Isono, 2020; Jung et al., 2020; Wu et al., 2021). In the model plant *Arabidopsis thaliana*, nine *ATG8* genes were identified and classified into three separate groups. Intriguingly, the *AtATG8h-i* group have a characteristic C-terminal exposed glycine residue that does not require ATG4 protease-dependent cleavage prior to their lipidation (Seo et al., 2016; Kellner et al., 2017). Although the analysis of the *At*ATG8 gene family is incomplete, the expression of several *At*ATG8 genes showed different yet partially overlapping patterns (Sláviková et al., 2005; Boycheva Woltering and Isono, 2020), supporting that different ATG8s share redundant roles while individual ATG8 members may have distinct and specific functions. Therefore, it remains challenging to distinguish the impact of each ATG8 isoform merely based on characterization of single knockouts due to functional redundancy.

Although most of the plant *ATG* genes are expressed at a ubiquitous and basal level, they can be induced by various developmental cues and environmental stimuli (Yoshimoto et al., 2004; Sláviková et al., 2005; Thompson et al., 2005; Rose et al., 2006; Peng et al., 2007; Chung et al., 2009; Avin-Wittenberg et al., 2018; Rodriguez et al., 2020; Qi et al., 2021). Ectopic overexpression of certain *ATGs* in plants successfully upregulated autophagy for plant fitness and stress tolerance (Xia et al., 2012; Li et al., 2015; Wang et al., 2016; Wang et al., 2017a; Wang et al., 2017b; Avin-Wittenberg et al., 2018; Minina et al., 2018; Sun et al., 2018a; Sun et al., 2018b). Compared to the extensive identification of transcription factors (TFs) regulating *ATGs* in animal and yeast cells, only a few TFs were discovered for their role in activation or repression of *ATGs* in plants. In cassava, WRKY20 was identified as a transcriptional activator of *ATG8a* (Yan et al., 2017). In nitrogen (N)-starved tomato leaves, the brassinosteroid (BR)-activated TF BRASSINAZOLE-RESISTANT1 (BZR1) binds to the promoters of *ATG2* and *ATG6* and induces autophagosome formation (Wang et al., 2019). The tomato heat shock TF HsfA1a was shown to upregulate the expression of *ATG10* and *ATG18f* and thereby inducing autophagy for drought tolerance (Wang et al., 2015). Recently, a study using yeast one-hybrid (Y1H) screening has revealed the binding of 225 TFs to the promoter of several *AtATG8s* (Wang et al., 2020). However, only the basic leucine-zipper protein TF TGA9 was further validated to transcriptionally upregulate the expression of *AtATG8b* and *AtATG8e* (Wang et al., 2020).

Inorganic phosphate (Pi) is an essential nutrient to plants for their growth and reproduction, but is poorly accessible to plants in most soils (Manning, 2008). To cope with the low availability of Pi, plants acquire a series of metabolic and morphological strategies, including enhancing Pi acquisition and remobilization, increasing exudation of organic acid and phosphatase, and remodeling of root architecture (Crombez et al., 2019; Wang et al., 2021; Paz-Ares et al., 2022). Several TFs were identified to be responsible for the regulation of Pi starvation-responsive (PSR) genes (Jain et al., 2012). Among them, PHOSPHATE STARVATION RESPONSE1 (PHR1) has been extensively studied and shown to act as a master regulator of PSR genes (Rubio et al., 2001; Bustos et al., 2010). In *Arabidopsis*, nearly 2,000 PSR genes are controlled by PHR1, perhaps *via* binding to the PHR1-binding sites (P1BS) (Castrillo et al., 2017). Although PHR1 is weakly transcriptionally responsive to low Pi stress, its activity is regulated by the nuclear SPX (SYG1/Pho81/XPR1) domain proteins (Bari et al., 2006; Puga et al., 2014; Wang et al., 2014). Moreover, an increased number of lateral roots is often regarded as a typical adaptive response to Pi limitation in *Arabidopsis* and in species that produce cluster roots (Desnos, 2008; Crombez et al., 2019). Such phenotypic change may generate a greater number of root tips to enlarge the potential hotspots for Pi uptake (Kanno et al., 2016). Nevertheless, the results from many other studies in *Arabidopsis* as well as in other species were occasionally in disagreement with the increased lateral root response upon Pi starvation (Crombez et al., 2019).

Compared to the wealth of investigations on carbon (C) and N starvation-induced autophagy (Avila-Ospina et al., 2014; Havé et al., 2017), the mechanism by which plant cells sense Pi limitation and induce autophagy is relatively less explored. An early study using tobacco BY-2 cells expressing aggregate-prone fluorescent proteins showed that Pi deprivation induced autophagy to remove the aggregates (Toyooka et al., 2006; Tasaki et al., 2014). Recent analysis of GFP-*At*ATG8a-labeled autophagic structures also suggested that low P induced the autophagosome formation in *Arabidopsi*s root tips and such responses were exaggerated in the *pdr2* but attenuated in the *pdr2/ire1a* mutants, thereby linking Pi limitation-induced autophagy to the ER stress-dependent signaling pathway (Naumann et al., 2019). In addition, when Pi limitation was combined with a reduced C/N ratio, Rubisco-containing body (RCB)-mediated chlorophagy was induced (Yoshitake et al., 2021). Our recent study revealed that low Pi preferentially increased the autophagic flux in the differential zone of the *Arabidopsis* root and most *AtATG* genes are highly induced by N starvation but moderately upregulated by Pi starvation (Chiu et al., 2023). Among the *AtATG8* family, *AtATG8a*, *AtATG8f*, *AtATG8g* and *AtATG8h* were upregulated by Pi starvation in the shoot, but only *AtATG8f* and *AtATG8h* were strikingly upregulated in the Pi-deprived root (Chiu et al., 2023). In this study, we further investigated the Pi starvation-induced transcriptional regulation of *AtATG8f* and *AtATG8h* and their spatial expression patterns. We also explored the physiological implication of Pi starvation-induced upregulation of *AtATG8f* and *AtATG8h*. Characterization of the *atg8f*/*atg8h* double mutants showed that loss of *At*ATG8f and *At*ATG8h reduces the autophagic activities of root under Pi starvation but does not affect the cellular Pi levels. In addition, the *atg8f*/*atg8h* double mutants exhibited decreased number of lateral roots under both Pi-replete and Pi-deplete conditions but not under N-starved conditions. Although Pi starvation-induced upregulation of *AtATG8f* and *AtATG8h* is PHR1-dependent, the results of Y1H and dual luciferase analyses indicated that PHR1 may not directly transactivate these two genes. As *AtATG8f* and *AtATG8h* are strongly expressed in the root stele tissues and involved in the lateral root development, we hypothesize that PHR1 may act upstream of *At*ATG8f and *At*ATG8h to fine-tune the root cell type-specific autophagic activity under Pi starvation.

## Results

### Pi deficiency induces the expression of *AtATG8f* and *AtATG8h* in a *At*PHR1-dependent manner

**Figure 1:**
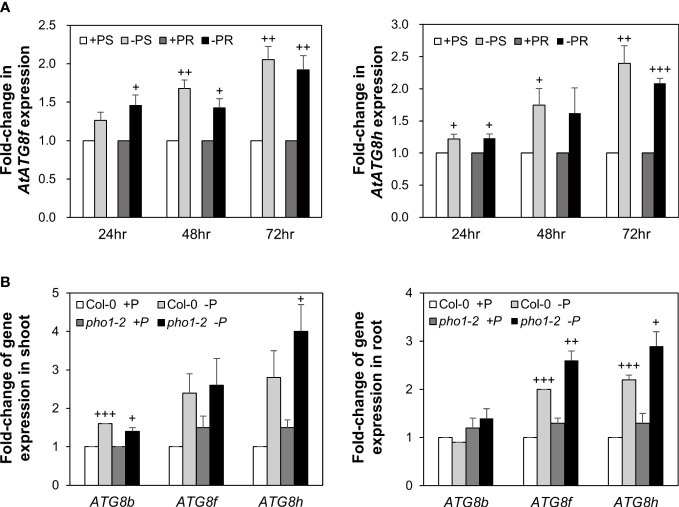
Low Pi induction of *AtATG8f* and *AtATG8h* is progressive and exacerbated in the *pho1-2* mutant. **(A)** Fold-change of expression of *AtATG8f* and *AtATG8h* in the shoot (S) and root (R) of 11-day-old *Arabidopsis* WT seedling following 24, 48 and 72 hours of Pi starvation (–P, 0 µM KH_2_PO_4_) conditions as determined by qRT-PCR. Error bars represent SE (n = 3, biological replicate pools of 20 seedlings collected from three independent experiments). ^+++^P< 0.001 ^++^P< 0.01, ^+^P< 0.05 compared to Pi-sufficient conditions; Student’s *t*-test; two-tailed. **(B)** Fold-change of expression of *AtATG8f* and *AtATG8h* expression in the shoot and root of 11-day-old *Arabidopsis* WT and *pho1-2* seedling under Pi-sufficient (+P, 250 µM KH_2_PO_4_) and Pi-deficient (–P, 0 µM KH_2_PO_4_, 3 days of starvation) conditions as determined by qRT-PCR. *AtATG8b* expression was used for comparison. Error bars represent SE (n = 3, biological replicate pools of 20 seedlings collected from three independent experiments). ^+++^P< 0.001 ^++^P< 0.01 ^+^P< 0.05 compared to Pi-sufficient conditions within the same genotype; Student’s *t*-test; two-tailed.

**Figure 2:**
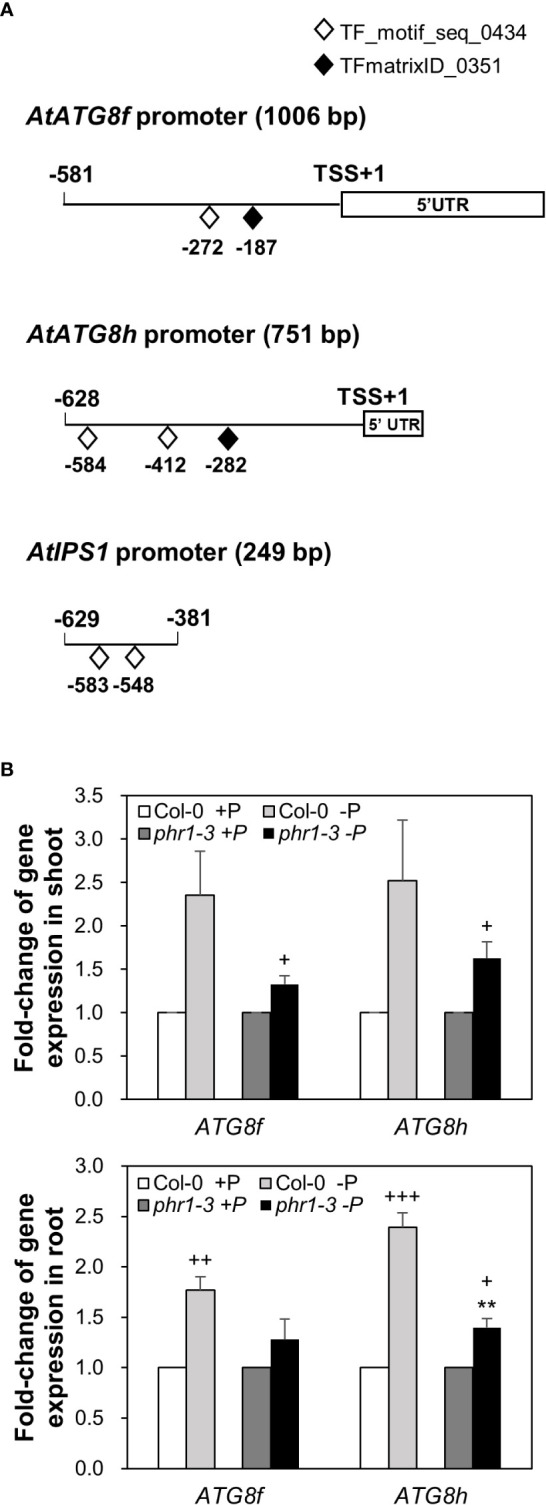
PHR1-dependent Pi starvation-induced upregulation of *AtATG8f* and *AtATG8h.* **(A)** Putative PHR1 binding sites (P1BS) predicted by the matrix TF_motif_seq_0434 (white diamond) and TFmatrixID_0351 (black diamond) in the proximal promoter of *AtATG8f, AtATG8h*, and *AtIPS1*. TSS, transcription start site; UTR, untranslated region. **(B)** Fold-change of expression of *AtATG8f* and *AtATG8h* in the shoot and root of 11-day-old *Arabidopsis* WT and *phr1-3* seedlings grown under Pi-sufficient (+P, 250 µM KH_2_PO_4_) and Pi-deficient (–P, 0 µM KH_2_PO_4_, 3 days of starvation) conditions as determined by qRT-PCR. Error bars represent SE (n = 3, biological replicate pools of 20 seedlings collected from three independent experiments). ^+++^P< 0.001 ^++^P< 0.01 ^+^P< 0.05 compared to Pi-sufficient conditions within the same genotype; ^**^P< 0.01, compared to Pi-deficient WT; Student’s *t*-test; two-tailed.

Our recent study has revealed that Pi limitation upregulated the expression of *AtATG8f* and *AtATG8h* among the *ATG8* family (Chiu et al., 2023). We further monitored the expression of these two genes in the wild-type (WT) plants at 24-, 48-, and 72-hour time points following Pi deprivation as well as in the *pho1-2* mutant, which exhibits extremely low shoot levels of Pi (Poirier et al., 1991). The progressive increase of *AtATG8f* and *AtATG8h* transcripts during Pi limitation (**Figure 1A**) as well as the exacerbated upregulation of *AtATG8f* and *AtATG8h* in the shoot and/or root of *pho1-2* under Pi limitation (**Figure 1B**) suggested that *AtATG8f* and *AtATG8h* are induced according to the magnitude of Pi deficiency. We were then prompted to determine which TFs are involved in such upregulation. To find out whether *AtATG8f* and *AtATG8h* could be upregulated by *At*PHR1, we set out to search for potential *cis*-elements in the promoter region of *AtATG8f* and *AtATG8h* that may be recognized by *At*PHR1. By using the PlantPan3.0 server (Chow et al., 2019), we found two and three putative P1BS elements in the proximal promoter of *AtATG8f* and *AtATG8h*, respectively (**Figure 2A**; **Table S1**). To validate whether *At*PHR1 participates in the regulation of *AtATG8f* and *AtATG8h*, we examined the expression of *AtATG8f* and *AtATG8h* in the *phr1-3* mutant (Rubio et al., 2001; Ren et al., 2012). The Pi starvation upregulation of *AtATG8f AtATG8h* was suppressed in both the shoot and root of *phr1-3* (**Figure 2B**), indicating that Pi limitation induces the expression of *AtATG8f* and *AtATG8h* in a PHR1-dependent manner.

### *At*PHR1 does not directly transactivate the expression of *AtATG8f* and *AtATG8h* in *Arabidopsis* mesophyll protoplasts

**Figure 3:**
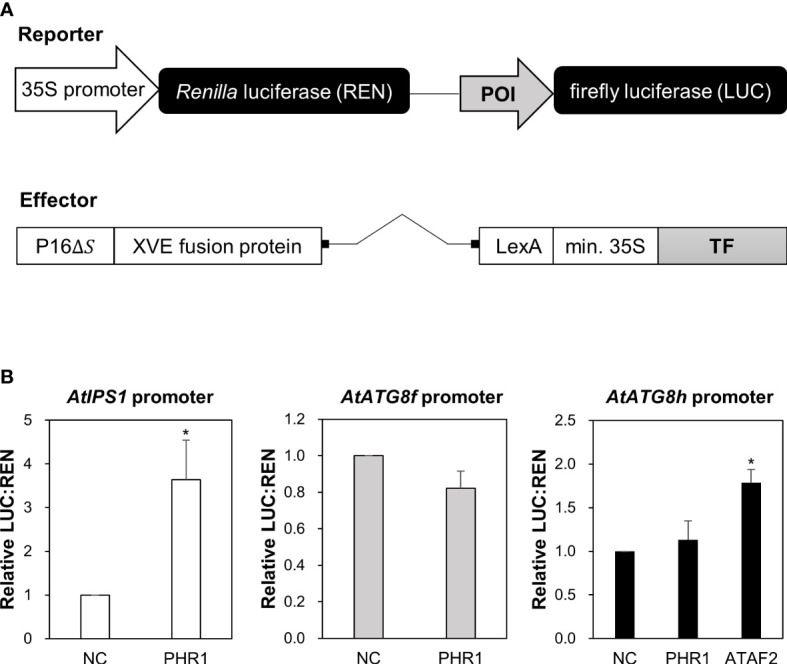
Transactivation of *AtATG8f* and *AtATG8h* promoters not by *At*PHR1 in *Arabidopsis* mesophyll protoplasts. **(A)** Schematic design of the reporter and effector constructs used for dual-luciferase assay (not drawn in scale). 35S promoter: CaMV 35S promoter; POI, promoter of interest; P16ΔS: a constitutive promoter; XVE: a chimeric transcription activator; LexA: an operator; min.35S: minimal 35S promoter; TF, transcription factor. **(B)** The relative LUC: REN ratios for the co-expression of the reporter construct containing the *AtIPS1*, *AtATG8f*, or *AtATG8h* promoter with the effector construct containing the transcription factor *At*PHR1 or *At*ATAF2. The co-expression of the effector construct expressing GFP and the corresponding reporter construct was taken as the negative control (NC). Data represent mean ± S.E. of biological replicates from independent experiments (n = 4 for the *AtATG8f* promoter and n = 3 for the *AtIPS1* and *AtATG8h* promoters). ^*^P< 0.05, compared to NC; Student’s *t*-test; two-tailed.

In our initial attempt to search for potential TFs that bind to the promoter region of *AtATG8f* and *AtATG8h* by Y1H, we surprisingly failed to identify *At*PHR1 as a positive candidate (**Figure S1**). In parallel, we performed transient dual-luciferase reporter assays using *Arabidopsis* mesophyll protoplasts to test whether *At*PHR1 transactivates the expression of *AtATG8f* and *AtATG8h in planta*. For the reporter constructs encoding firefly luciferase (LUC) and *Renilla* luciferase (REN), the genomic sequences of each promoter were cloned into the pGreenII-0800-Luc vector (Hellens et al., 2005) (**Figure 3A**). For the effector construct, we used the ß-estradiol-inducible XVE expression system in the pGPTVII backbone to express TFs (Schlücking et al., 2013) (**Figure 3A**). In addition, the reporter construct carrying the promoter sequences of *AtIPS1* containing two P1BS elements was used as the positive control (**Figure 3A**) (Bustos et al., 2010). When *At*PHR1 was co-expressed with *P_IPS1_*:LUC/*P_35S_*:REN, the ratio of LUC : REN was increased to 2.9-fold as compared to the negative control in which GFP was co-expressed (**Figure 3B**). When we co-expressed the NAC domain TF *At*ATAF2 as a positive control with *P_ATG8h_*:LUC/*P_35S_*:REN (Wang et al., 2020), the ratio of LUC : REN was increased by 1.8-fold (**Figure 3B**). In comparison, when *At*PHR1 was co-expressed with *P_ATG8f_*:LUC/*P_35S_*:REN or *P_ATG8h_*:LUC/*P_35S_*:REN, the ratio of LUC : REN was similar to that of the GFP control (**Figure 3B**). These results indicated that *At*PHR1 may not directly transactivate *AtATG8f* and *AtATG8h.*

### Loss of *At*ATG8f and *At*ATG8h does not impair cellular Pi homeostasis

**Figure 4:**
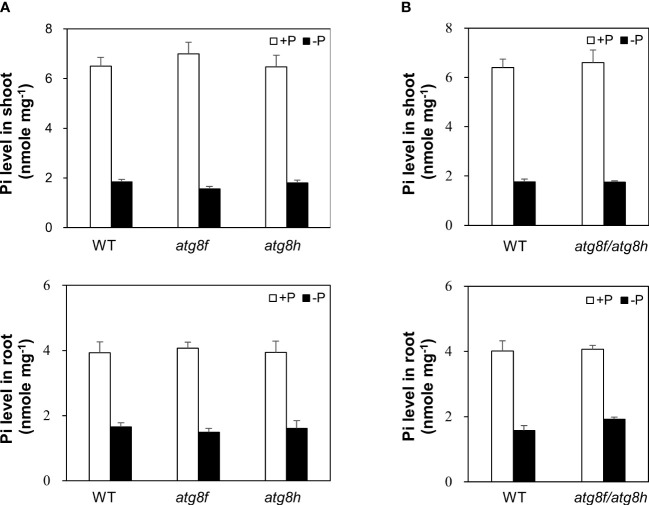
Pi levels of *atg8f*, *atg8h* and *atg8f/atg8h* mutants. **(A, B)** The shoot and root Pi levels of 11-day-old *Arabidopsis* seedlings of WT, *atg8f*, and *atg8h* **(A)** and *atg8f/atg8h* **(B)** under Pi-sufficient (+P, 250 µM KH_2_PO_4_) and Pi-deficient (–P, 0 µM KH_2_PO_4_, 3 days of starvation) conditions. Error bars represent SE (n = 9, biological replicate pools of 10 seedlings collected from three independent experiments).

To investigate the physiological role of *AtATG8f* and *AtATG8h*, we obtained the homozygous T-DNA lines for each gene: *atg8f-2*, *atg8f-3*, *atg8f-5*, and *atg8f-6* for *AtATG8f* and *atg8h-2* and *atg8h-3* for *AtATG8h* (**Figure S2**; **Table S2**). By reverse transcription polymerase chain reaction (RT-PCR), we validated that the full-length transcripts of *AtATG8f* were absent in the *atg8f-2* and *atg8f-5* homozygotes (**Figure S2**), indicating that these two mutants carry null alleles. We only chose *atg8f-5* (hereafter referred to as *atg8f*) for further study because the T-DNA insertion site in this mutant was closer to the 5’ untranslated region (UTR) of *AtATG8f*, which likely resulted in complete disruption of the transcription. On the other hand, the full-length transcripts of *AtATG8h* were not detected in both the *atg8h-2* and *atg8h-3* mutants. Nevertheless, we were able to detect some truncated transcripts in *atg8h-2* (**Figure S2**), and therefore *atg8h-3* (hereafter referred to as *atg8h*) was used. Through crosses we also successfully generated the *atg8f-5/atg8h-3* double mutant (hereafter referred to as *atg8f/atg8h*). The expression of *AtATG8f* and *AtATG8h* was induced in the WT Pi-starved roots but not detectable in *atg8f/atg8h* under both Pi-replete and Pi-deplete conditions (**Figure S2**). Of note, the transcript expression of the other *AtATG8* genes was comparable in WT and *atg8f/atg8h* (**Figure S3**), suggesting no compensatory upregulation of the other *AtATG8* members for the loss of *AtATG8f* and *AtATG8h* in the double mutant. To investigate whether *At*ATG8f and *At*ATG8h could be involved in the maintenance of cellular Pi homeostasis, we measured the shoot and root Pi levels of *atg8f, atg8h*, and *atg8f/atg8h*. All of them showed no difference from WT under both Pi-replete and Pi-deplete conditions (**Figures 4A, B**), suggesting that defective *AtATG8f* and *AtATG8h* do not affect cellular Pi levels.

### *At*ATG8f and *At*ATG8h account for the maintenance of autophagic flux in the root under Pi starvation

**Figure 5:**
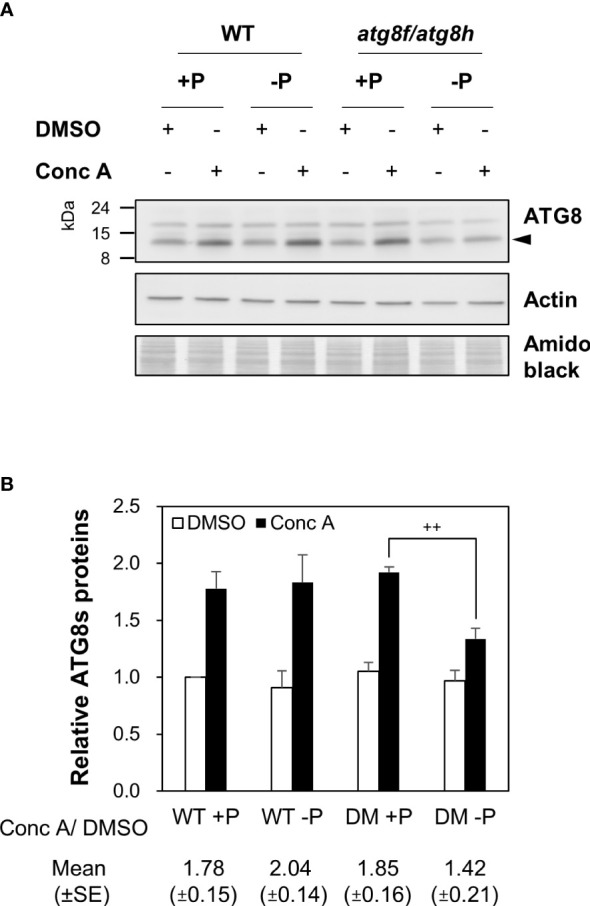
*AtATG8f* and *AtATG8h* are required for the maintenance of autophagic flux in the Pi-starved roots. **(A)** Immunoblot analysis of the expression of ATG8s in the root of 11-day-old *Arabidopsis* WT and *atg8f/atg8h* seedlings under Pi-sufficient (+P, 250 µM KH_2_PO_4_) and Pi-deficient (–P, 0 µM KH_2_PO_4_, 3 days of starvation) conditions with or without Conc A treatment (1 µM, 6 h). Representative images are shown. Arrowhead indicates the bands of ATG8s. **(B)** The expression change of ATG8s and the autophagic flux in the root of *Arabidopsis* WT and DM (*atg8f/atg8h*) seedlings. The expression level of ATG8s was normalized with the corresponding actin. Error bars represent SE (n = 3, biological replicate pools of 20 seedlings collected from three independent experiments). ^++^P< 0.01, compared to Pi-sufficient DM with Conc A treatment; Student’s *t*-test; two-tailed. The relative autophagic flux was calculated by dividing the normalized ATG8s signal intensity of Conc A-treated samples by that of DMSO controls. Amido black staining was used for total protein detection.

To evaluate whether the low Pi induction of *AtATG8f* and *AtATG8h* may change autophagic activities, we attempted to compare autophagic flux between WT and *atg8f/atg8h*. The GFP-ATG8 cleavage assay is a widely accepted tool to measure autophagic flux by calculating the ratio of the amount of cleaved GFP to the amount of full-length GFP-ATG8 (Klionsky et al., 2021). However, this approach would unfortunately introduce additional ATG8s into *atg8f/atg8h*. We therefore conducted the ATG8 degradation assay to estimate the autophagic flux in the root of *atg8f/atg8h*. As the steady-state abundance of ATG8s can be influenced by autophagy activation or blockage of downstream steps such as inefficient vacuolar fusion or decreased degradation (Zhang et al., 2013), the vacuolar H^+^-ATPase inhibitor concanamycin A (Conc A) was applied to prevent ATG8s from vacuolar degradation (Dröse et al., 1993; Bowman and Bowman, 2005). Without the availability of *At*ATG8f and *At*ATG8h-specific antibodies, we performed immunoblotting with a polyclonal anti-ATG8s antibody that recognize all the *At*ATG8 isoforms (ATG8s). Regardless of Pi status, ATG8s were found to accumulate in the WT root upon Conc A treatment (**Figure 5A**). In the absence of Conc A, the abundance of ATG8s in the total root proteins was comparable in *atg8f/atg8h* and WT (**Figure 5A**). This may be because only a small proportion of ATG8s were contributed by *At*ATG8f and *At*ATG8h transcripts (**Figure S3**). Nonetheless, the relative autophagic flux in the WT root calculated based on the changes of ATG8s between DMSO control and Conc A treatment showed no differences between Pi-replete and Pi-depleted conditions (**Figure 5B**). These results were in good agreement with our recent findings (Chiu et al., 2023). Notably, the autophagic flux was comparable in the Pi-repleted root of *atg8f/atg8h* and WT but reduced in the Pi-depleted root of *atg8f/atg8h* (**Figures 5A, B**). Given that the abundance of membrane-associated ATG8s would correlate with autophagic activity, we then compared the amount of ATG8s in the root microsomal fraction between WT and *atg8f/atg8h*. While the microsomal-enriched ATG8s was missing in the autophagy-defective *atg7-3* mutant, it was slightly reduced in the Pi-deplete root of WT (**Figure S4**). There was a substantial decrease of microsomal-enriched ATG8s in the root of *atg8f/atg8h* as compared to WT, but no significant difference was found between Pi-replete and Pi-deplete root of *atg8f/atg8h* (**Figure S4**). We then further examined ATG8s lipidation in WT and *atg8f/atg8h* by immunoblot. Because to distinguish lipidated ATG8s from non-lipidated ATG8s using immunoblot analyses was reported to be technically challenging due to the multiple variants in plants (Yoshimoto et al., 2004; Chung et al., 2010), we applied phospholipase D (PLD) treatment, which hydrolyzes the terminal phosphodiester bonds of phospholipids to produce phosphatidic acid (PA). The PLD-mediated cleavage of ATG8-PE yields ATG8-ethanolamine and PA, thus helping identify bands that correspond to lipidated ATG8s. The lipidated ATG8s migrated faster than the unmodified form during SDS-PAGE in the presence of urea and were sensitive to PLD digestion and absent in the *atg5* and *atg7* backgrounds (Yoshimoto et al., 2004; Chung et al., 2010; Suttangkakul et al., 2011; Li et al., 2014; Zhuang et al., 2017; Luo and Zhuang, 2018). Our results indicated that Pi starvation did not change the abundance of lipidated ATG8s in the WT root, but the lipidated ATG8s was unexpectedly increased in the Pi-replete root of *atg8f/atg8h* and remained a similar level or slightly declined following Pi starvation (**Figure S5**).

Besides ATG8s, NBR1 known as a selective autophagy receptor is itself a substrate degraded in the vacuole (Svenning et al., 2011; Zhou et al., 2013; Ji et al., 2020; Jung et al., 2020). Disruption of *At*NBR1 conferred increased sensitivity to heat, drought, and salt stresses (Zhou et al., 2013; Ji et al., 2020). However, *At*NBR1 does not play an essential role in regulating N deprivation-induced autophagy (Lin et al., 2020). To answer whether *At*NBR1 is involved in Pi starvation-induced autophagy and thus its degradation could be used as an alternative method for measuring autophagic flux in the root, we monitored the expression changes of *At*NBR1 in the WT root following 12, 24, 48, 72 hours of Pi deprivation. The specificity of anti-NBR1 antibodies was validated with the *nbr1-2* and *atg7-3* mutants by the absence and accumulation of *At*NBR1 proteins, respectively (**Figure S6**). Either with 6 or 12 hours of Conc A treatment, *At*NBR1 accumulated in the WT root to a similar extent at different time point of Pi starvation (**Figure S6**). Of note, the expression changes of *At*NBR1 in the WT root upon Conc A treatment appeared to be smaller than that of ATG8s (**Figures 5A**, **S6**). It is possible that *At*NBR1 is subjected to selective autophagic degradation only under certain stress conditions. Accordingly, Pi deprivation did not alter the vacuolar degradation of *At*NBR1 in the WT root (**Figure S6**). There was also no difference of *At*NBR1 degradation between *atg8f/atg8h* and WT (**Figure S7**), indicating that *At*NBR1 may not participate in Pi starvation-induced autophagy. Overall, these results revealed that *At*ATG8f and *At*ATG8h contribute to a substantial proportion of microsomal-enriched ATG8s and may regulate the autophagic flux under Pi starvation through a mechanism other than promoting ATG8s lipidation.

### Expression of *AtATG8f* and *AtATG8h* in the root stele and at the sites where lateral root primordia develop

**Figure 6:**
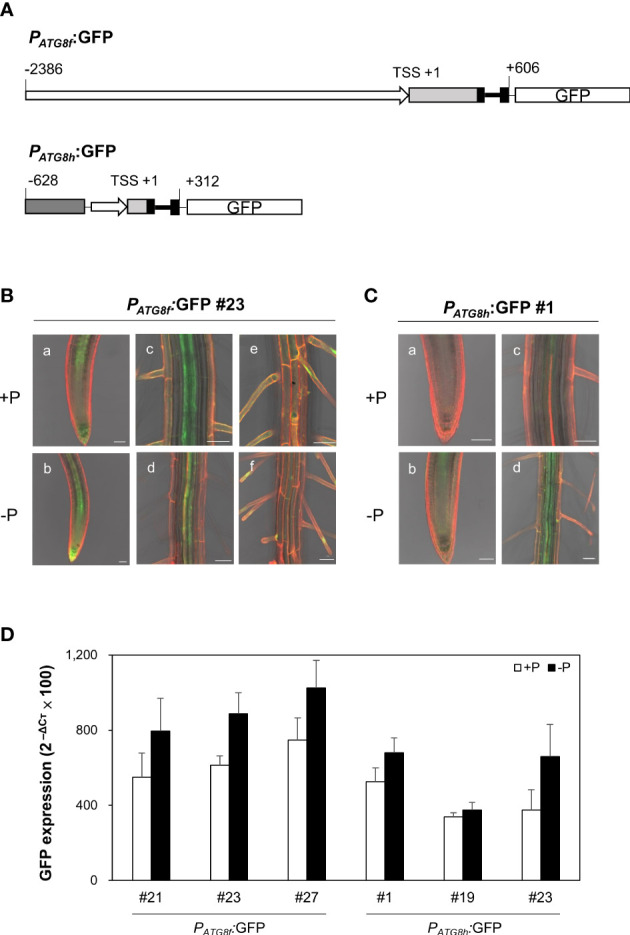
Expression patterns of *AtATG8f* and *AtATG8h* in *Arabidopsis* GFP reporter lines. **(A)** Schematic design of *AtATG8f* and *AtATG8h* promoter-fused GFP reporter constructs. The upstream region of the putative transcription starts site (TSS) in *AtATG8f* and *AtATG8h* were indicated by rightwards thick arrows. Black, light gray and dark gray boxes represent exons, 5´UTR and the gene At3g06430, respectively. Thick and thin lines indicate introns and linkers, respectively. The schematic structure is drawn according to scale. **(B, C)** GFP expression in the root of 3-day-old seedlings of *P_ATG8f_*:GFP **(B)** and *P_ATG8h_*:GFP **(C)** germinated under Pi-sufficient (a, c, and e; +P, 250 μM KH_2_PO_4_) and Pi-deficient (b, d, and f; –P, 0 μM KH_2_PO_4_) conditions. GFP signals in the root apical meristem (a, b), the vascular tissue **(C, D)** and the root hair **(E, F)**. Scale bars = 50 μm. At least two independent lines were examined for each construct and representative images are shown. Propidium iodide (PI) was used as a root cell wall stain. **(D)** qRT-PCR analysis of GFP expression in the root of 11-day-old *P_ATG8f_*:GFP and *P_ATG8h_*:GFP seedlings grown under Pi-sufficient (+P, 250 µM KH_2_PO_4_) and Pi-deficient (–P, 0 µM KH_2_PO_4,_ 3 days of starvation) conditions. Error bars represent SE (n = 3, biological replicate pools of 20 seedlings from three independent experiments).

To examine the spatial expression patterns of *AtATG8f* and *AtATG8h* under Pi starvation, we generated GFP reporter lines, designated *P_ATG8f_*:GFP and *P_ATG8h_*:GFP. The promoter sequence of *AtATG8f* we used starts from 2386 bp upstream of the putative transcription start site (TSS) to 606 bp downstream of the TSS within the second exon as shown (**Figure 6A**). This is much longer than the one used by Di Berardino et al., which contains the 1651 bp upstream of the TSS and the 176 bp downstream of the TSS (Di Berardino et al., 2018). The upstream region of the TSS in our construct is also longer than the one used by Sláviková et al., which includes the 1906 bp upstream of ATG codon plus the entire coding regions of *AtATG8f*, a total of 3125-bp genomic sequence containing the exons and introns (Sláviková et al., 2005). While the study of Di Berardino et al. indicated the expression of *AtATG8f* in the veins of the pericarp and in the seed embryo, the study of Sláviková et al. displayed the expression of *AtATG8f* in the root of seedlings with relatively poor resolution at the cell-type level. As for *AtATG8h*, due to the short intergenic region between *AtATG8h* and the upstream gene At3g06430, two *AtATG8h* promoter regions were considered in our study. The shorter one contains a total of 553 bp, starting from 221 bp upstream of the TSS to 312 bp downstream of the TSS. The longer one contains the partial genomic sequences of At3g06430 and extending to 312 bp downstream of the TSS within the second exon (**Figure 6A**). Overall, there were no differences in the expression levels and patterns of GFP between the *AtATG8h* reporter lines with different promoter lengths (data not shown), so we chose the transgenic lines with the longer *AtATG8h* promoter for our further investigation. Confocal analysis of the root of *P_ATG8f_*:GFP lines showed that the expression of *AtATG8f* was in the root apical meristem, root cap, stele tissues, and root hairs of the primary root under Pi sufficiency (**Figure 6B**). By comparison, the GFP signals in *P_ATG8h_*:GFP lines were much weaker and mainly detected in the root stele tissues (**Figure 6C**). Of note, GFP signals were hardly detected in the root cap and root hairs of *P_ATG8h_*:GFP lines under Pi sufficiency (**Figure 6C**). Under Pi deficiency, the GFP expression patterns of *P_ATG8f_*:GFP and *P_ATG8h_*:GFP lines were similar as those under Pi sufficiency and the signals in the root hair showed stronger intensities (data not shown). Further quantitative real-time PCR analysis of GFP expression in the Pi-starved root of *P_ATG8f_*:GFP and *P_ATG8h_*:GFP lines also supported the upregulation of GFP expression by low Pi (**Figure 6D**), which was in good agreement with the increased endogenous *AtATG8f* and *AtATG8h* transcripts in these reporter lines (**Figure S8**). These results suggested that *AtATG8f* and *AtATG8h* can be upregulated by Pi starvation at the transcriptional level.

**Figure 7:**
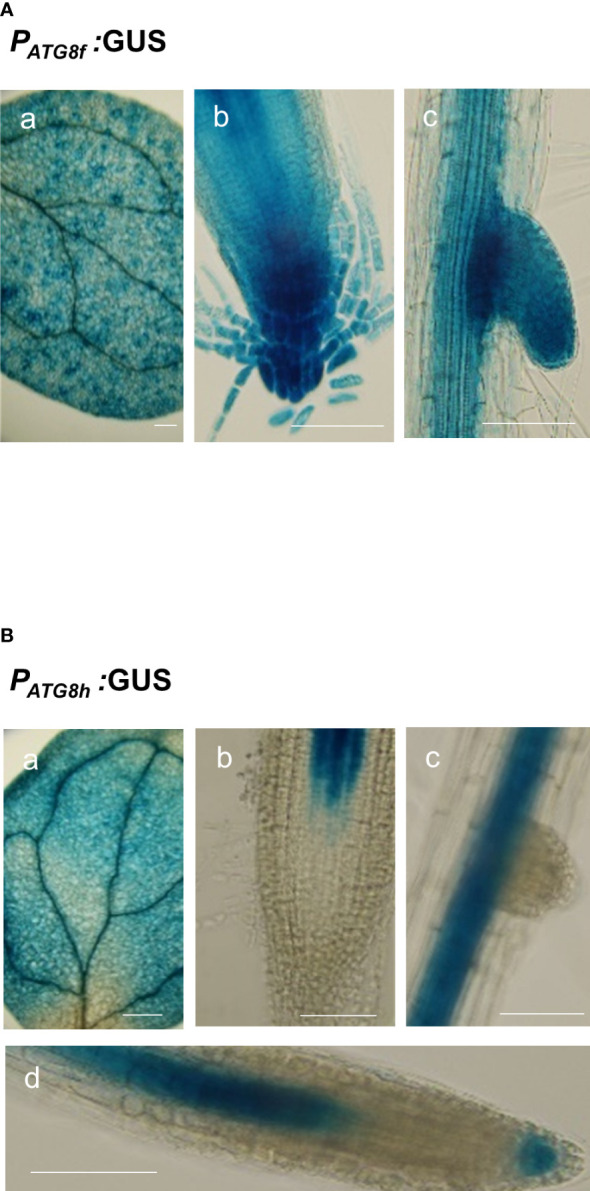
Expression patterns of *At*ATG8f and *At*ATG8h in *Arabidopsis* GUS reporter lines. **(A, B)** GUS staining in the 8-day-old seedlings of *P_ATG8f_*:GUS **(A)** and *P_ATG8h_*:GUS **(B)** seedlings under full nutrient (+PN, 250 μM KH_2_PO_4_ and 7.5 mM NO_3-_) conditions. GUS signals in the cotyledon (a), root apical meristem (b), lateral root primordia (c), and lateral root tip (d). Scale bars, 50 μm. The time of GUS staining for *P_ATG8f_*:GUS and *P_ATG8h_*:GUS, was 1 and 2 hours, respectively. At least two independent lines were examined for each construct and representative images are shown.

To assess the promoter activities of *AtATG8f* and *AtATG8h* in the shoot, we also generated *P_ATG8f_*:GUS and *P_ATG8h_*:GUS lines, in which the promoter sequences used were the same as those used in the GFP lines. The expression of *AtATG8f* was mainly found in the shoot vascular tissues and mesophylls (**Figure 7A**). Similarly, the GUS staining of *P_ATG8h_*:GUS lines was predominantly in the similar shoot tissues yet with weaker signals (**Figure 7B**). Moreover, the *P_ATG8f_*:GUS lines showed the expression patterns of *AtATG8f* in the root stele tissues of both primary and lateral roots as well as in fully emerged lateral root primordia (**Figure 7A**). Of note, the promoter activity of *AtATG8f* was detected throughout the development of lateral root (**Figure 7A**), which was consistent with *P_ATG8f_*:GFP lines (**Figure S9**). By comparison, the promoter activity of *AtATG8h* was absent in the primary root apical meristem but detectable in the basal meristem (**Figure 7B**). Importantly, the GUS staining of *AtATG8h* reporter lines was neither detectable in the lateral root primordia nor at early stages of lateral root development (**Figure 7B**). Only after the establishment of lateral root meristem, we could detect the expression of *AtATG8h* in the stele and columella of lateral root (**Figure 7B**), which was also consistent with *P_ATG8h_*:GFP lines (**Figure S9**).

### Loss of *At*ATG8f and *At*ATG8h suppresses the lateral root number

**Figure 8:**
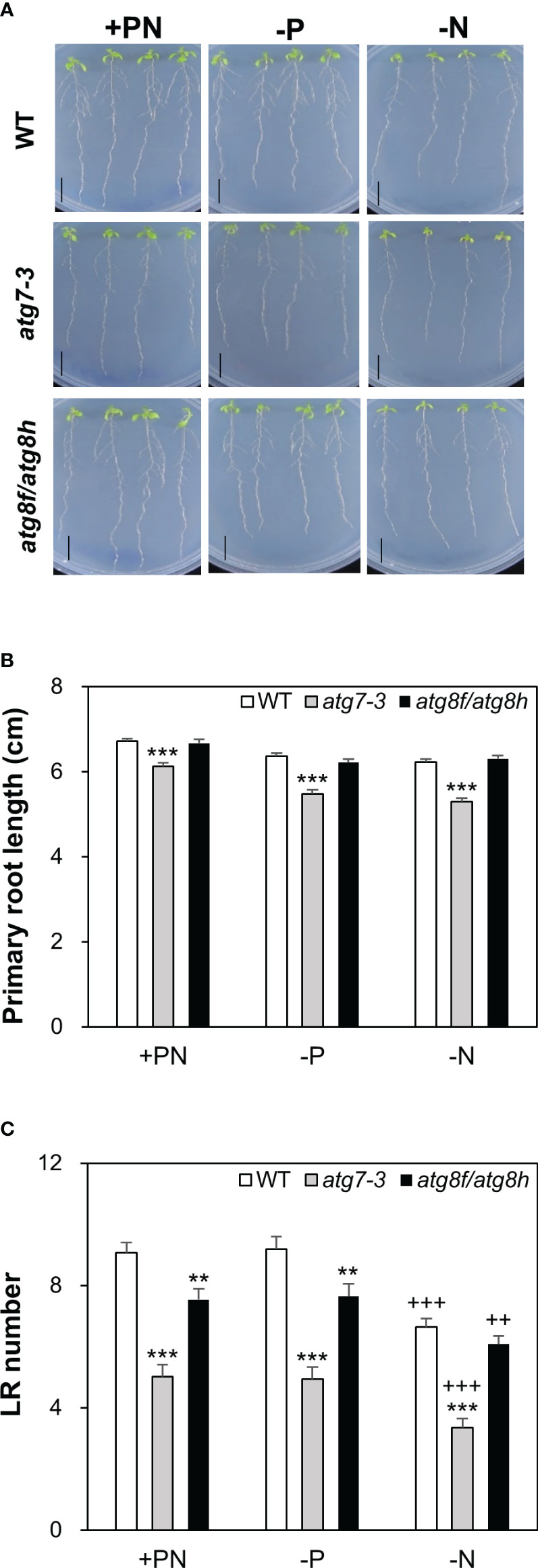
Loss of *At*ATG8f and *At*ATG8h suppresses lateral root development. **(A)** Representative images of 10-day-old WT, *atg7-3*, and *atg8f/atg8h* seedlings under full nutrient (+PN, 250 μM KH_2_PO_4_ and 7.5 mM ${\text{NO}}_{3}^{-}$ ), Pi-deficient (–P, 0 μM KH_2_PO_4_, 5 days of starvation) and N-deficient (–N, 0.1 mM ${\text{NO}}_{3}^{-}$ , 5 days of starvation) conditions. Scale bars = 1 cm. **(B, C)** Data are presented for the primary root (PR) length **(B)** and the lateral root (LR) number **(C)** of WT, *atg7-3* and *atg8f/atg8h* seedlings. Error bars represent SE (n = 35–40, collected from three independent experiments). ^+++^P< 0.001 ^++^P< 0.01, compared to Pi-sufficient conditions within the same genotype; ^***^P< 0.001 ^**^P< 0.01, compared to WT under the same conditions; Student’s *t*-test; two-tailed.

Next, we focused to characterize the root phenotypes of *atg8f/atg8h* mutants and used the *atg7-3* mutant for comparison (Huang et al., 2019). Pi starvation is known to induce the synthesis of extracellular acid phosphatases and organic acids for P mobilization (Marschner, 1995). Considering that the phytochemical or metabolite crosstalk between plants under nutrient deficiency may affect the root phenotypes of different genotypes when grown on the same plate, we grew four seedlings for each genotype *per* plate to avoid the mutual effect of root exudates from different genotypes. Under our full nutrient and Pi- and N-deprived conditions, the primary root length showed no difference between WT and *atg8f/atg8h* but was shorter in *atg7-3* (**Figures 8A, B**). These results suggested that unlike the impairment of the single-copy *ATG* gene, loss of *At*ATG8f and *At*ATG8h does not retard the primary root growth. Because strong *AtATG8f* and *AtATG8h* expression was observed during the lateral root development, we set out to analyze the number of lateral roots for *atg8f/atg8h*. Similar to the results of previous studies showing the inhibition of lateral growth under severe N starvation (Krouk et al., 2010; Gruber et al., 2013), we observed a reduction of lateral root number *per* seedling in all genotypes grown on N-limited media (**Figure 8C**). The lateral root number was strikingly reduced in the autophagy-defective *atg7-3* mutant under all the growth conditions, implying that functional autophagy is required for the lateral root development (**Figure 8C**). Intriguingly, the lateral root number was also significantly reduced in the *atg8f/atg8h* relative to the WT under Pi-rich and Pi-starved conditions (**Figure 8C**), indicating that *ATG8f* and *ATG8h* are involved in the regulation of lateral root growth. While under N starvation the lateral root number of *atg7-3* was reduced relative to the WT, no significant differences were found between WT and *atg8f/atg8h* (**Figure 8C**), indicating that the other *At*ATG8 may share redundant roles in lateral root development during N starvation.

## Discussion

### PHR1 acts upstream of the transcriptional regulation of *AtATG8f* and *AtATG8h*

A chromatin immunoprecipitation sequencing (ChIP-seq) study has revealed *AtATG8f* to be a direct target of *At*PHR1 (Castrillo et al., 2017), while a previous Y1H screen discovered that *At*PHR1 was not among the 32 TFs interacting with the *AtATG8h* promoter (Wang et al., 2020). In our Y1H assay, we failed to identify *At*PHR1 as a positive TF binding to the promoter of *AtATG8f* and *AtATG8h* (**Figure S1**). Results of our dual luciferase reporter assays also did not support a direct transactivation of *AtATG8f* and *AtATG8h* by *At*PHR1. Recently, a chromatin remodeling analysis of *Arabidopsis* Pi-starved roots suggested that *At*PHR1 activates a set of TFs triggering a second wave of epigenetic changes required for upregulation of PSR genes (Barragán-Rosillo et al., 2021). Intriguingly, the association of *AtATG8h* with increased chromatin accessibility (upDARs) was found in Pi-limited root of WT but not *phr1/phl2* (Barragán-Rosillo et al., 2021) (**Table S3**), indicating that PHR1 and/or its paralogues may engage transcriptional activation of *AtATG8h* in response to Pi limitation by enhancing chromatin accessibility. According to the ChIP-seq data (Castrillo et al., 2017), we found that only less than 20% of PHR1/PHL2-dependent Pi starvation-induced genes are direct targets of PHR1 (Barragán-Rosillo et al., 2021). This could in part explain the discrepancy among conclusions due to different methods or test systems. Nevertheless, it warrants further investigation as to whether other TFs are responsible for the transcriptional regulation of *AtATG8f* and *AtATG8h* under Pi limitation and whether low Pi induction of *AtATG8f* and *AtATG8h* involves the coordination of epigenetic and transcriptional changes.

### *AtATG8f* and *AtATG8h* finetune the autophagic flux in response to Pi starvation

ATG8 itself is degraded together with cargos and serves as a faithful proxy for autophagy activity readout (Klionsky et al., 2021). In this study, we showed that the expression changes of endogenous ATG8s were not prominent in the Pi-depleted root of WT and *atg8f/atg8h* relative to their respective Pi-replete controls (**Figures 5A, B**). This may be explained, at least in part, by the modest upregulation of *AtATG8f* and *AtATG8h* in certain root cell types. It is also likely that the abundance of the other ATG8s masks the small expression changes of *At*ATG8f and *At*ATG8h by Pi starvation (**Figure S3**). On the other hand, we could detect the reduction of microsomal-enriched ATG8s in the root of *atg8f/atg8h*, indicating that loss of *At*ATG8f and *At*ATG8h indeed decreased the abundance of membrane-associated ATG8s (**Figure S4**). Intriguingly, neither the autophagic flux nor the *At*ATG8s lipidation was increased by Pi starvation in the WT root, which reinforced the view of the two recent studies that nutrient starvation-induced autophagy is likely tissue- or cell type-specific (Dong et al., 2022; Chiu et al., 2023). While based on the expression changes of ATG8s between DMSO control and Conc A treatment, the relative autophagic flux was reduced in the Pi-starved root of *atg8f/atg8h* (**Figures 5A, B**), the autophagic flux estimated by NBR1 degradation failed to support this conclusion (**Figure S7**). We thought that NBR1 may not play a direct role in Pi starvation-induced autophagy, at least at the whole-root level. Nevertheless, we found the increased amount of lipidated ATG8s in the *atg8f/atg8h* root (**Figure S5**). The discrepancy between the reduced autophagic flux and the increased lipidated ATG8s in the Pi-depleted root of *atg8f/atg8h* might hint that the lipidation/de-lipidation of the other isoforms is altered and/or that *At*ATG8f and *At*ATG8h fine-tune the autophagic flux under Pi starvation through an unknown ATG8s lipidation-independent pathway. In mammalian cells, knockout of all the ATG8 family members suggested that the ATG8s are dispensable for autophagosome formation but crucial for autophagosome–lysosome fusion (Nguyen et al., 2016). However, it remains to discover whether the plant ATG8s are also important at this step to regulate the autophagic flux. It is worth noting that there are intrinsic limitations in measuring autophagic flux changes based on the steady-state abundance of ATG8s in the whole root. Even in the presence of vacuolar inhibitors that isolate autophagy induction from inhibition of autophagic degradation, this assay obscures estimates of substrate clearance – the most ideal measure of autophagic flux (Klionsky et al., 2021) and thus cannot determine the autophagic flux at the cellular level. The development of tool that allows to quantify autophagic responses at cell-type specific resolution as well as generation of *At*ATG8f and *At*ATG8h-specific antibodies may advance these issues (Stephani and Dagdas, 2020).

### Role of *AtATG8f* and *AtATG8h* in the lateral root development

In our recent study of *Arabidopsis* autophagy-defective mutants, we showed that the *atg5-1*, *atg7-3* and *atg10-1* mutants exhibited impaired Pi homeostasis and compromised plant fitness in response to fluctuating Pi availability (Chiu et al., 2023). However, we did not observe similar phenotypes for *atg8f, atg8h*, and *atg8f/atg8h* (**Figures 4A, B**). Regardless of nutrient conditions, the primary root length showed no difference between WT and *atg8f/atg8h* but was shorter in *atg7-3* (**Figures 8A, B**). Rather, we found a reduction in the lateral root number of both *atg7-3* and *atg8f/atg8h* (**Figure 8C**). The development of lateral root primordia is sensitive to the availability of N (Banda et al., 2019; Santos Teixeira and ten Tusscher, 2019). Under severe N starvation, the primary root length, the lateral root length, and the number of lateral roots *per* primary root were reported to be inhibited in *atg4a4b-1* (Yoshimoto et al., 2004). We also found that compared to WT, *atg7-3* but not *atg8f/atg8h* had a decreased lateral root number under relatively mild N deficiency (**Figure 8C**). We reasoned that while *At*ATG8f and *At*ATG8h are critical for lateral root development under full nutrient and Pi-starved conditions, some other ATG8s are induced under N limitation and thus compensate for the loss of *At*ATG8f and *At*ATG8h. It is known that nutrient cues can affect lateral root formation *via* crosstalk with hormone signaling at four key developmental steps: initiation, primordium establishment, emergence, and elongation (Jia et al., 2021). As *AtATG8f* is present throughout the lateral root formation and *AtATG8h* starts to express likely after vascular tissue differentiation, we speculate that *AtATG8f* and *AtATG8h* may be involved in the lateral root development at different stages, which needs to be further studied. Intriguingly, we found that *AtATG8f* but not *AtATG8h* is expressed in the root cap. The periodicity of lateral root formation is driven by programmed cell death of the root cap (Xuan et al., 2016). Prior to the root cap cell death, autophagy has been shown to be required for organelle clearance and organized cell separation (Goh et al., 2022). In addition, selective autophagy was previously proposed to promote the lateral root development upon Pi starvation through ARK2-PUB9 module-dependent auxin accumulation (Deb et al., 2014; Sankaranarayanan and Samuel, 2015). However, the underlying mechanism remains to be elucidated on a molecular basis.

## Materials and methods

### Plant material and growth conditions

Seeds of the *Arabidopsis thaliana atg7-3* (SAIL_11_H07), *atg8f-2* (SALK_052510C), *atg8f-3* (SALK_039231), *atg8f-5* (SALK_133008), *atg8f-6* (SALK_004370), *atg8h-2* (SALK_021495), *atg8h-3* (SALK_136493), *phr1-3* (SALK_067629), *nbr1-2* (GK-246H08), and *pho1-2* (Poirier et al., 1991) mutants used in this study were in the Columbia (Col) background and obtained from the Arabidopsis Biological Resource Center (ABRC). The *Arabidopsis* seeds were surface-sterilized and germinated on agar plates with one-half modified Hoagland’s solution containing 1% Suc and 0.8% Bacto agar (BD Difcom 204010), and grown in the growth chamber at 22°C with a 16 h light/8 h dark cycle. The full nutrient (+PN) or Pi-sufficient (+P) and Pi-deficient (−P) media were supplemented with 250 μM and 0 or 10 µM KH_2_PO_4_, respectively, unless specified otherwise. The full nutrient (+PN) and N-deficient (−N) media were supplemented with 7.5 mM and 0 or 0.1 mM µM Ca(NO_3_)_2_/KNO_3_, respectively, unless specified otherwise.

### Construct design

All the insert fragments of interest were amplified by polymerase chain reaction (PCR) and cloned into pJET1.2/blunt vector for sequencing and then subcloned into the desired vectors. For the constructs used for dual-luciferase assay, the promoter sequences of *AtATG8f*, *AtATG8h* and *AtIPS1* were subcloned into the pGreenII-0800-Luc vector (Hellens et al., 2005). The full-length coding sequences of *At*ATAF2 and *At*PHR1 were subcloned into the β-estradiol-inducible P16ΔS:sXVE:S10 vector (Liu et al., 2018). For the constructs used for Y1H analysis, the promoter sequences of *AtATG8f* and *AtATG8h* were as same as those used for dual-luciferase reporter constructs and were cloned into the pHISi2 vector in which extra start codons of pHISi (Clontech/Takara bio Inc.) residing within 5’ untranslated region of the reporter gene HIS3 are mutated. For the GFP or GUS reporter constructs, the *P_ATG8f_*:GFP or *P_ATG8h_*:GFP constructs were obtained by inserting the promoter sequences of *AtATG8f* and *AtATG8h* in the binary vector pMDC111. The *P_ATG8f_*:GUS or *P_ATG8h_*:GUS constructs were made by inserting the genomic sequences into the binary vector pMDC163. Primer sequences used for gene cloning are listed in **Table S4**.

### Yeast one-hybrid analysis

The yeast strain YM4271 was employed for Y1H analysis of the *AtATG8f* and *AtATG8h* promoters, which was performed as described previously (Mitsuda et al., 2010) but with some modifications. The promoter-cloned pHISi construct was linearized with the restriction enzyme ApaI (for *AtATG8f*) or NcoI (for *AtATG8h*) and the promoter::*HIS3* fusion was then integrated into the YM4271 genome. A total of 1,736 *Arabidopsis* transcription factor genes were cloned into pGADT7 vector (Clontech/Takara bio Inc.), divided into 384 mini pools and individual interactions between each promoter and mini pool were examined by the yeast growth on the selective media lacking leucine (L), uracil (U) or histidine (H) with or without the addition of 3-amino-1,2,4-triazole (3-AT) as indicated.

### *Arabidopsis* mesophyll protoplast isolation and transfection

Leaves of 4-week-old *Arabidopsis* plants grown under 12 h light/12 h dark were harvested and protoplasts were isolated following the tape-*Arabidopsis* sandwich method (Wu et al., 2009) with minor modifications. About 2.5×10^4^ cells were transfected by the PEG/calcium-mediated method (Yoo et al., 2007). An equal volume of the freshly-prepared PEG 4000 solution containing 40% (w/v) PEG, 0.1 M CaCl_2_, and 0.2 M mannitol was added, completely mixed, and incubated at RT for 10 min. A 600 μL of modified W5 solution (154 mM NaCl, 125 mM CaCl_2_, 5 mM KCl, 5 mM glucose, and 2 mM MES) was added and gently mixed to stop the transfection. Transfected protoplasts were collected by centrifugation at 100 g for 2 min and were re-suspended in 0.5 mL of W5 solution. The final protoplasts were incubated in a 1% BSA pre-coated 12-well plate at 22°C for 16 hours in light. 10 μg/mL (36.7 μM) *β*-estradiol in ethanol was added 8 hours before performing the dual-luciferase assay.

### Dual-luciferase assay in *Arabidopsis* protoplasts

Dual-luciferase assays were carried out as described with slight modifications (Hellens et al., 2005). Briefly, after 8 hours of induction, the transfected protoplast suspension was transferred to a 1.5 mL centrifugation tube and centrifuged at 100 g for 10 min. The supernatant was discarded and the pellets were re-suspended in 100 μL of 1X passive lysis buffer (PLB) provided in the Dual Luciferase Reporter Assay System kit (Promega). Protoplasts were disrupted by vortex for 10 s followed by centrifugation at 10,000 g for 2 min. A 5 μL of the supernatant sample was loaded into a well of a white flat bottom Costar 96 well plate (Corning). Dual-luciferase assays were performed in Synergy™ HTX Multi-Mode Microplate Reader (BioTek). A 40 μL luciferase assay reagent and a 40 μL Stop and Glo reagent (Promega) were injected *per* well. The ratio of LUC to REN was measured to represent the activity of the corresponding promoter when the effector plasmid DNA was co-transfected.

### Phosphate concentration analysis

Pi concentrations were analyzed as described (Ames, 1966) with minor modifications. For the measurement of Pi concentrations, fresh tissue was frozen with liquid nitrogen and homogenized with 1% glacial acetic acid and incubated at 42°C for 30 min followed by centrifugation at 13,000 g for 5 min. The supernatant aliquot was mixed with the assay solution (0.35% NH_4_MoO_4_, 0.86 N H_2_SO_4_, and 1.4% ascorbic acid) and incubated at 42°C for 30 min. Pi content determined by colorimetric assay based on the formation of phosphomolybdate was measured at A_750_.

### RNA isolation, reverse transcription PCR, quantitative real-time RT-PCR

Total RNA from samples was isolated using GENEzol™ TriRNA Pure Kit with DNase (Geneaid, GZXD200). The first strand cDNA was synthesized from 0.5 μg total RNA using PrimeScript™ 1st strand cDNA Synthesis Kit (TaKaRa, 6110A) with oligo(dT) primer. qRT-PCR was performed using KAPA SYBR^®^ FAST qPCR Master Mix (2X) Kit on StepOnePlus™ Real-Time PCR System (Applied Biosystems) according to the manufacturer’s instructions. Relative expression levels were normalized to that of an internal control *ACT8* (At1g49240). Sequences of primers used are listed in **Table S5**.

### Immunoblot analysis

For extraction of total root protein, the roots of WT and mutant seedlings were ground in liquid nitrogen and dissolved in protein lysis buffer containing 60 mM 2‐amino‐2‐(hydroxymethyl)‐1,3‐propanediol (Tris)-HCl (pH 8.5), 2% Sodium dodecyl sulfate (SDS), 2.5% glycerol, 0.13 mM EDTA, 1 mM phenylmethylsulfonyl fluoride (PMSF) and Protease Inhibitor Cocktail (Sigma-Aldrich P9599). A total of 25 µg root protein from each sample was loaded onto 12% Q-PAGE™ Bis-Tris Precast Gel (SMOBIO) or NuPAGE 4–12% Bis-Tris Gels (Thermo Fisher Scientific) and transferred to polyvinylidene difluoride (PVDF) membranes. The membrane was blocked with 1 or 2% BSA in 1X PBS solution with 0.2% Tween 20 (PBST, pH 7.2) at room temperature for 1 h and hybridized with primary antibodies of ATG8 (1:1000; Agrisera AS14 2811), NBR1 (1:4000; Agrisera AS14 2805) and actin (1:4000; Abcam, ab197345) for 1 h at room temperature in blocking solution. The membrane was washed four times with 1X PBST for 5 min followed by hybridization with the horseradish peroxidase–conjugated secondary antibody (1:10,000–20,000 dilution; GeneTex GTX213110-01) in blocking solution for 1 h. After four washes in 1× PBST for 5 min and a rinse with distilled water, chemiluminescent substrates (Advansta, WesternBright ECL) for signal detection were applied.

### Isolation of root microsomal protein and ATG8 lipidation assay

Root microsomal protein was isolated with the Minute Plant Microsomal Membrane Extraction Kit (Invent, MM-018) according to the manual instruction. The resultant pellets (microsomal protein) were resuspended in the solubilization buffer containing 350 mM sucrose, 0.5% Triton X-100, 10 mM Tris-MES (pH 7.0), 1 mM Dithiothreitol (DTT) and Protease Inhibitor Cocktail (Sigma-Aldrich P9599). A total of 10 µg root microsomal protein from each sample was loaded onto NuPAGE 4–12% Bis-Tris Gels (Thermo Fisher Scientific) and transferred to PVDF membranes for further immunoblot analysis as described above, except with 3% BSA-containing blocking solution. Phospholipase D (PLD; Enzo Lifesciences BML-SE301) treatment was performed by mixing 10 µg root microsomal protein with 80 U PLD in reaction buffer containing 10 mM Tris-HCl (pH 8.0), 1% glycerol, 0.01% Triton X-100 and incubated at 37°C for 1 h. Each sample was loaded onto 15% mPAGE^®^ TurboMix Bis-Tris Gel (TMKIT, Merck) with 6 M urea for electrophoresis according to the manual instruction and transferred to PVDF membranes for further immunoblot analysis as described above.

### *Arabidopsis* transformation and transgenic plant selection

The binary plasmid was introduced into *A. tumefaciens* strain GV3101:pMP90 and selected on 5 μg ml^-1^ rifampicin, 50 μg ml^-1^ gentamycin and 50 μg ml^-1^ kanamycin. The *Arabidopsis* plants were transformed using standard floral dip method, and T1 transgenic plants were selected on half-strength MS medium supplemented with 1% sucrose plates containing appropriate antibiotics. T2 transgenic lines with a segregation ratio of 3 resistant: 1 sensitive were used for further study as presumably having single insertion of T-DNA.

### GUS staining

GUS activity was detected as previously described with modifications (Jefferson et al., 1987). Briefly, seedlings were placed in 90% acetone on ice after sampling and vacuum infiltrated in freshly prepared GUS assay buffer containing 500 mM NaH_2_PO_4_, 500 mM Na_2_HPO_4_ 7H_2_O, 1mM K_3_Fe(CN)_6_, 1 mM K_4_Fe(CN)_6_, 10 mM EDTA, 0.1% Triton X-100, and 2.25 mM X-Gluc (5-bromo-4-chloro-3-indoyl-β-D-glucuronide sodium salt; Cyrusbioscience) for 20 min followed by incubation at 37°C, 1 and 2 hours for *P_ATG8f_*:GUS and *P_ATG8h_*:GUS reporter lines, respectively. Destaining was made with ethanol to remove chlorophyll. GUS staining was observed under the stereomicroscope and Leica DM2000 microscope.

### Confocal microscopy

Confocal microscopy images were acquired using Zeiss LSM 800 with objectives Plan-Apochromat 40x/1.3 Oil DIC M27 in multi-track mode with line switching and averaging of two – four readings. The excitation/emission wavelengths for GFP and propidium iodide (PI) were 488 nm/530 nm and 548 nm/561 nm, respectively.

### Analysis of root morphology

Seedlings were germinated on one-half modified Hoagland’s media containing full nutrient (+PN) for 5 days and then transferred for vertical growth under full nutrient (+PN), Pi-deficient (0 μM KH_2_PO_4_) or N-deficient (0.1 mM ${\text{NO}}_{3}^{-}$ ) conditions for another 5 days. For each independent experiment, the plates were prepared with the same volume of medium from the same batch. For the lateral root analyses, at least 9 plates were taken for the total sample collection. Photos were taken by PowerShot G16 Camera. The length of the primary roots and the number of lateral roots with length longer than 0.25 cm *per* seedlings were calculated or counted using ImageJ (Schneider et al., 2012).

### Chemical treatments

The Concanamycin A (Conc A; 1 mM; Cayman 11050) and Acetosyringone (150 mM; Sigma-Aldrich D134406) stock solutions were prepared in dimethyl sulfoxide (DMSO). The PI working solution (20 µg/ml) was prepared from the stock solution (1 mg/ml; Invitrogen P3566). A six-hour of 1µM Conc A or DMSO treatment was applied in the sample preparation for immunoblot analysis of ATG8s and NBR1 proteins. β-estradiol (36.7 mM; Sigma-Aldrich E2758) and acetosyringone (150 mM; Sigma-Aldrich D134406) stock solutions were prepared in ethanol and DMSO, respectively.

## Data availability statement

The raw data supporting the conclusions of this article will be made available by the authors, without undue reservation.

## Author contributions

T-YL designed the research. L-YL, H-XC, C-HC, W-CC, T-YL and NM performed experiments. T-YL, L-YL, H-XC, C-HC and NM analyzed data. T-YL, L-YL, H-XC and NM wrote the manuscript. All authors contributed to the article and approved the submitted version.
