# Supplementary material for: Role of autophagy-related proteins ATG8f and ATG8h in the maintenance of autophagic activity in Arabidopsis roots under phosphate starvation
Source: Front Plant Sci. 2023 Jun 26;14:1018984. doi: 10.3389/fpls.2023.1018984 (PMC10331476; doi:10.3389/fpls.2023.1018984)
Supplement: Supplementary file 1 [file DataSheet_1.pdf]

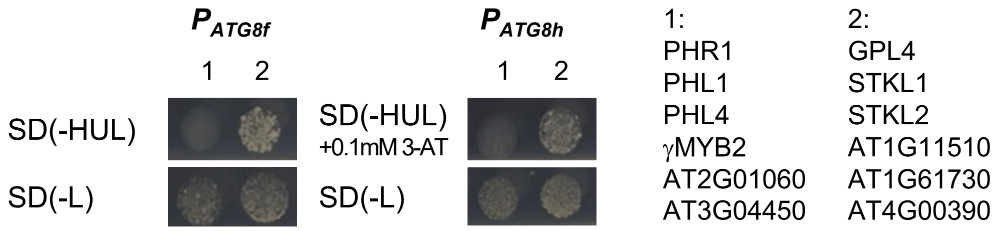

Supplemental Fig. 1 Y1H analysis of the binding of *At*PHR1 and other TFs to the promoter of *AtATG8f* and *AtATG8h*

SD (-HUL) is a selective medium in which yeast can grow if the transformed transcription factor binds to the promoter region. 3-AT stands for 3-amino-1,2,4-triazole which suppresses yeast growth driven by the background activity of each promoter. SD (-L) is a growth control medium in which any yeast with transgene should grow normally. Spots 1 and 2 represent the mixtures of the TFs listed on the right panel. Cells in spot 1 containing *At*PHR1 failed to grow on SD (-HUL) media, while cells in spot 2 grew well due to the binding of one or more of the expressed TFs to the promoter.

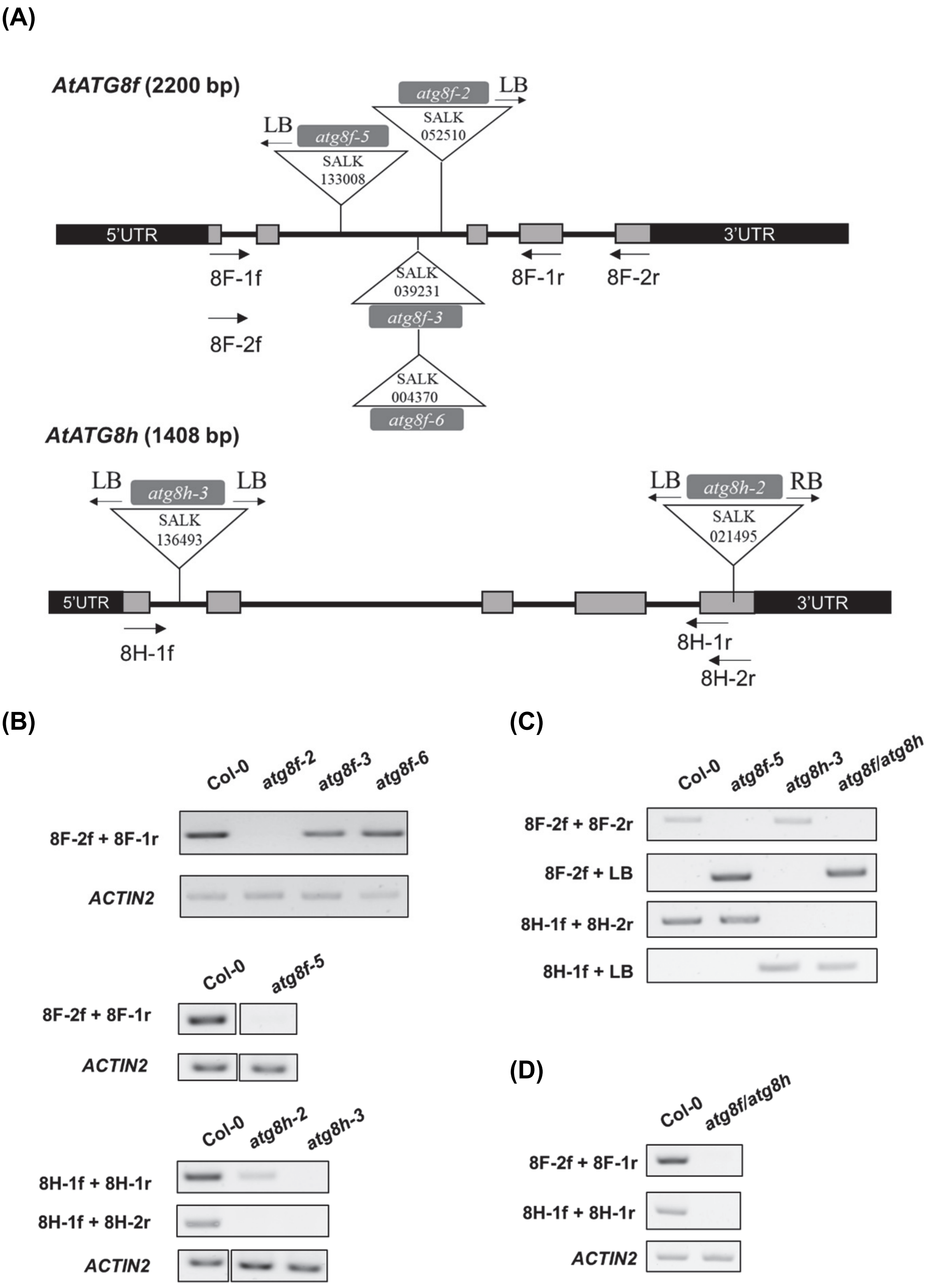
(A)
AtATG8f (2200 bp)
atg8f-2
LB
SALK
052510
LB
atg8f-5
SALK
133008
5'UTR
3'UTR
8F-1f
8F-1r
8F-2r
SALK
039231
atg8f-3
8F-2f
SALK
004370
atg8f-6
AtATG8h (1408 bp)
LB
atg8h-3
LB
SALK
136493
LB
atg8h-2
RB
SALK
021495
5'UTR
3'UTR
8H-1f
8H-1r
8H-2r
(B)
Col-0
atg8f-2
atg8f-3
atg8f-6
8F-2f + 8F-1r
ACTIN2
Col-0
atg8f-5
8F-2f + 8F-1r
ACTIN2
Col-0
atg8h-2
atg8h-3
8H-1f + 8H-1r
8H-1f + 8H-2r
ACTIN2
(C)
Col-0
atg8f-5
atg8h-3
atg8f/atg8h
8F-2f + 8F-2r
8F-2f + LB
8H-1f + 8H-2r
8H-1f + LB
(D)
Col-0
atg8f/atg8h
8F-2f + 8F-1r
8H-1f + 8H-1r
ACTIN2

Supplemental Fig. 2 Characterization of *AtATG8f* and *AtATG8h* T-DNA insertion mutants

(A) T-DNA insertion SALK lines for *AtATG8f* and *AtATG8h* alleles. The genomic structures of *AtATG8f* and *AtATG8* are shown. Black box, gray box, and black line represent the UTR, exon, and intron, respectively. The position of the T-DNA insertion is indicated by a triangle. The primers used for genotyping are indicated by arrows.

(B) Reverse transcription-polymerase chain reaction (RT-PCR) analysis of *atg8f-2*, *atg8f-3*, *atg8f-5*, *atg8f-6*, and *atg8h-2*, *atg8h-3* mutants. The primer set used for each amplicon is as indicated. *ACTIN2* was used as a loading control.

(C) Genotyping of *atg8f-5/atg8h-3* (*atg8f/atg8h*) mutants. The primer set used for each amplicon is as indicated.

(D) RT-PCR analysis of *atg8f/atg8h* mutants. The primer set used for each amplicon is as indicated. *ACTIN2* was used as a loading control.

(A)

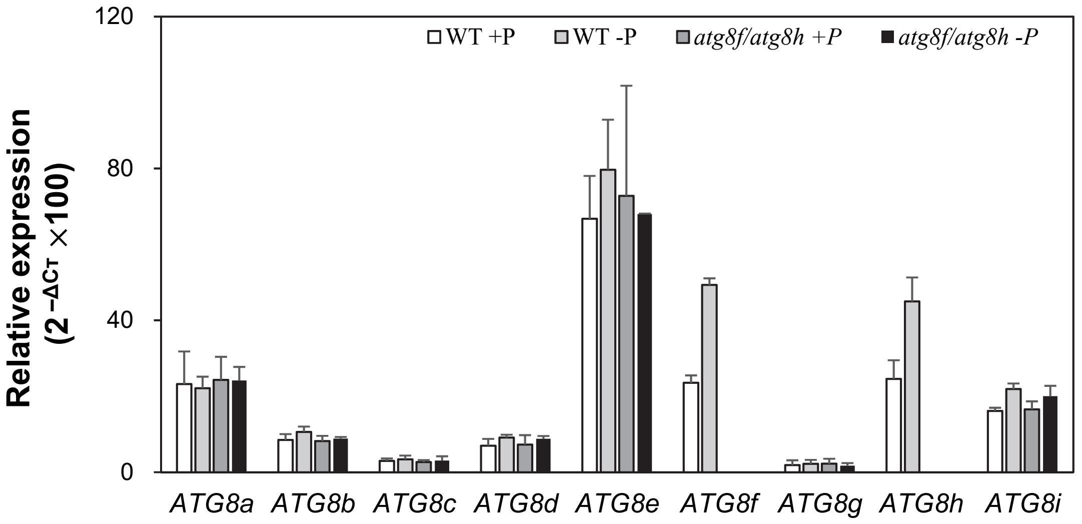

(B)

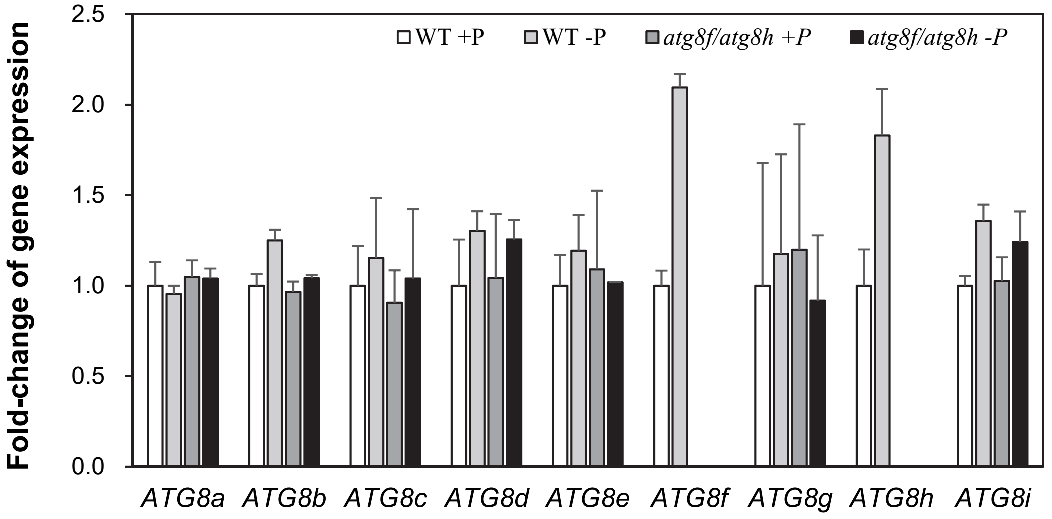

Supplemental Fig. 3 qRT-PCR analysis of *AtATG8s* expression in the root of WT and *atg8f/atg8h*

The relative expression (A) and fold change (B) of *AtATG8s* transcripts in the root of 11-day-old WT and *atg8f/atg8h* under Pi-sufficient (+P, 250 μM KH_2PO_4) and Pi-deficient (–P, 0 μM KH_2PO_4, 3 days of starvation) conditions. Error bars represent SE (n = 2, biological replicate pools of 20 seedlings collected from two independent experiments).

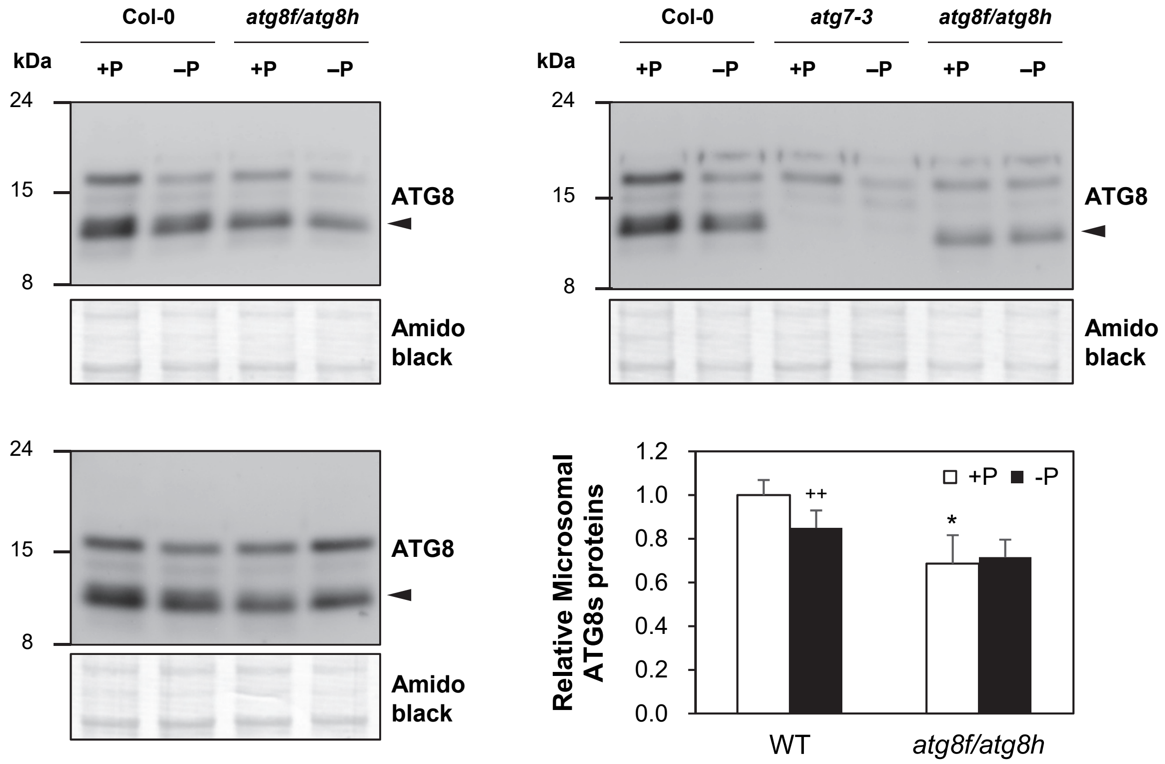

Supplemental Fig. 4 Immunoblot analysis of microsomal-enriched *At*ATG8s in the root of *atg8f/atg8h*

The expression of microsomal-enriched *At*ATG8s in the root of 11-day-old *Arabidopsis* WT, *atg7-3* and *atg8f/atg8h* under Pi-sufficient (+P, 250 µM KH_2PO_4) and Pi-deficient (–P, 0 µM KH_2PO_4, 3 days of starvation) conditions. Arrowhead indicates the bands of microsomal-enriched ATG8s. The images from three independent experiments were shown. The abundance of microsomal-enriched *At*ATG8s was normalized with the corresponding microsomal protein staining. Error bars represent SE (n = 3, biological replicate pools of seedlings collected from three independent experiments). $^{++}$, $P < 0.01$, compared to Pi-sufficient conditions; *, $P < 0.05$, compared to WT under Pi-sufficient conditions; Student's *t*-test; two-tailed. Amido black staining was used for protein detection.

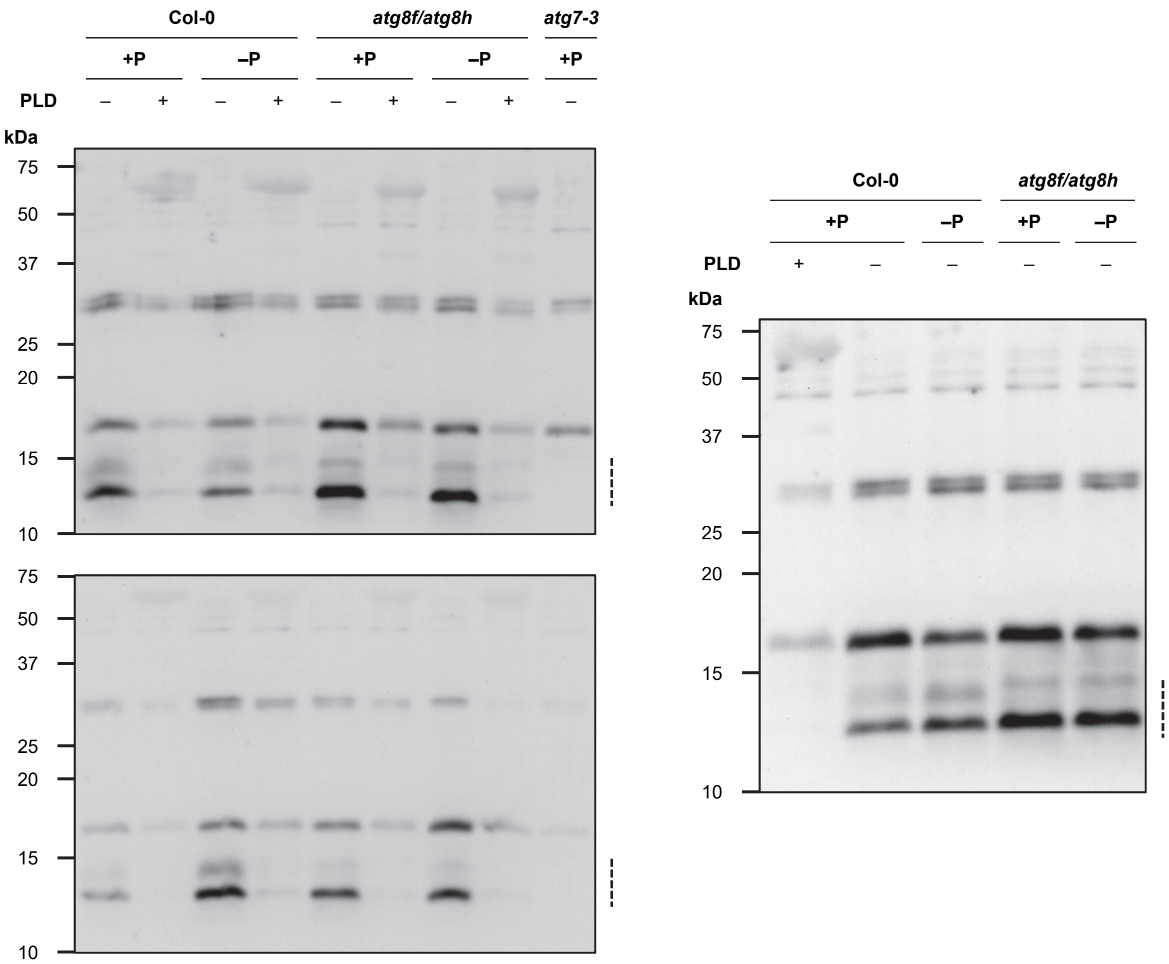

Supplemental Fig. 5 Immunoblot analysis of lipidated ATG8s in *Arabidopsis* root under Pi starvation

Detection of lipidated *At*ATG8s in the root of 11-day-old *Arabidopsis* WT, *atg8f/atg8h and atg7-3* under Pi-sufficient (+P, 250 μM KH_2PO_4) and Pi-deficient (–P, 0 μM KH_2PO_4, 3 days of starvation) conditions. Sample treated with phospholipase D (PLD) was shown as indicated. The dashed line locates the group of the possible lipidated ATG8s. The images from three independent experiments were shown.

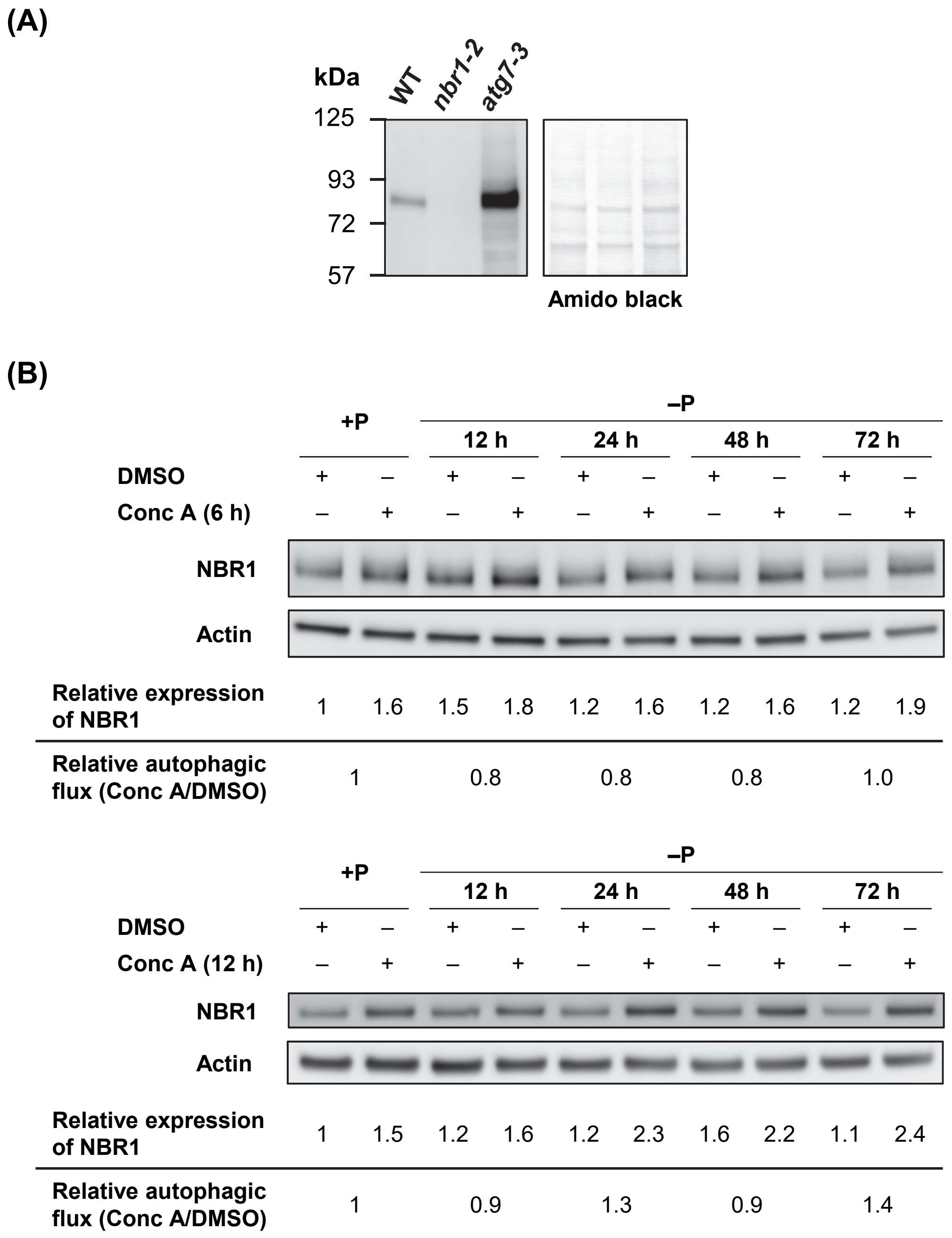

Supplemental Fig. 6 Protein expression of *At*NBR1 in Arabidopsis roots during Pi starvation

(A) Immunoblot detection of *At*NBR1 in the root of *Arabidopsis* WT, *nbr1-2* and *atg7-3* seedlings under Pi-sufficient (+P, 250 µM KH_2PO_4) conditions.

(B) Immunoblot analysis of *At*NBR1 in the root of 11-day-old *Arabidopsis* WT. Plants were grown under Pi-sufficient (+P, 250 µM KH_2PO_4) and Pi-deficient (–P, 12–72 hours of starvation, 0 KH_2PO_4) conditions treated with DMSO or 1 µM of Conc A (upper panel: 6 h; lower panel: 12 h). The expression level of endogenous NBR1 was normalized with the corresponding actin. The autophagic flux was calculated by dividing the normalized NBR1 signal intensity of Conc A-treated samples by that of DMSO controls. A biological replicate represents pools of 10 seedlings.

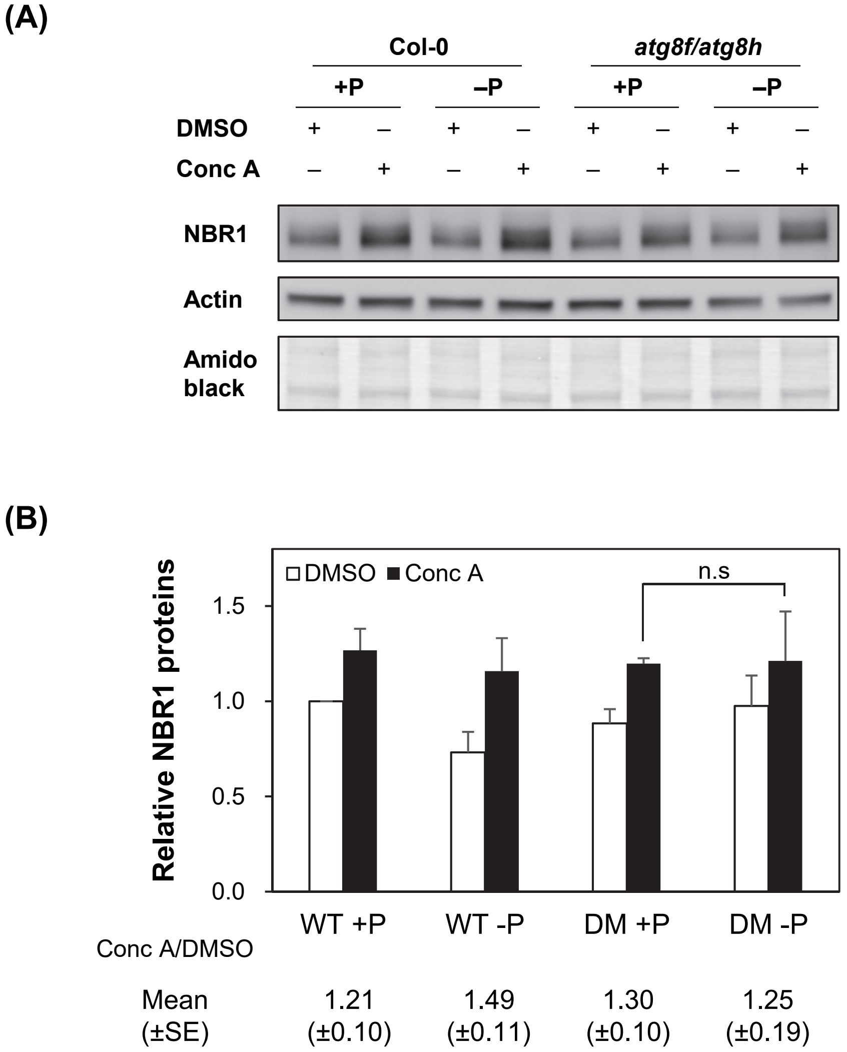

Supplemental Fig. 7 Degradation of *At*NBR1 in *Arabidopsis* roots during Pi starvation
(A) Immunoblot analysis of the expression of NBR1 in the root of 11-day-old *Arabidopsis* WT and *atg8f/atg8h* seedlings under Pi-sufficient (+P, 250 μM KH_2PO_4) and Pi-deficient (–P, 0 μM KH_2PO_4, 3 days of starvation) conditions with or without Conc A treatment (1 μM, 6 h). Representative images are shown.
(B) The expression change of NBR1 in the root of *Arabidopsis* WT and DM (*atg8f/atg8h*) seedlings. Error bars represent SE (n = 3, biological replicate pools of 10 seedlings collected from three independent experiments). n.s, non-significant; $P > 0.05$, compared to DM under Pi-sufficient conditions with Conc A treatment; Student's *t*-test; two-tailed. The expression level of NBR1 was normalized with the corresponding actin. The relative autophagic flux was calculated by dividing the normalized NBR1 signal intensity of Conc A-treated samples by that of DMSO controls. Amido black staining was used for total protein detection.

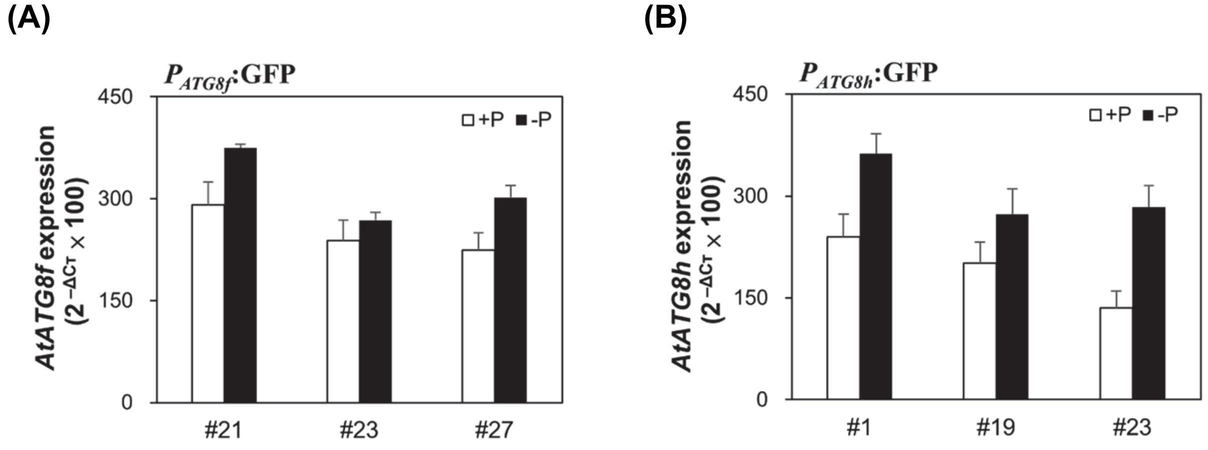

Supplemental Fig. 8 qRT-PCR analysis of endogenous *AtATG8f* and *AtATG8h* expression in the root of GFP reporter lines

The *AtATG8f* expression (A) and the *AtATG8h* expression (B) in the root of 11-day-old P_{ATG8f}:GFP and P_{ATG8h}:GFP, respectively under Pi-sufficient (+P, 250 μM KH_2PO_4) and Pi-deficient (–P, 0 μM KH_2PO_4, 3 days of starvation) conditions. Error bars represent SE (n = 3, biological replicate pools of 20 seedlings collected from three independent experiments).

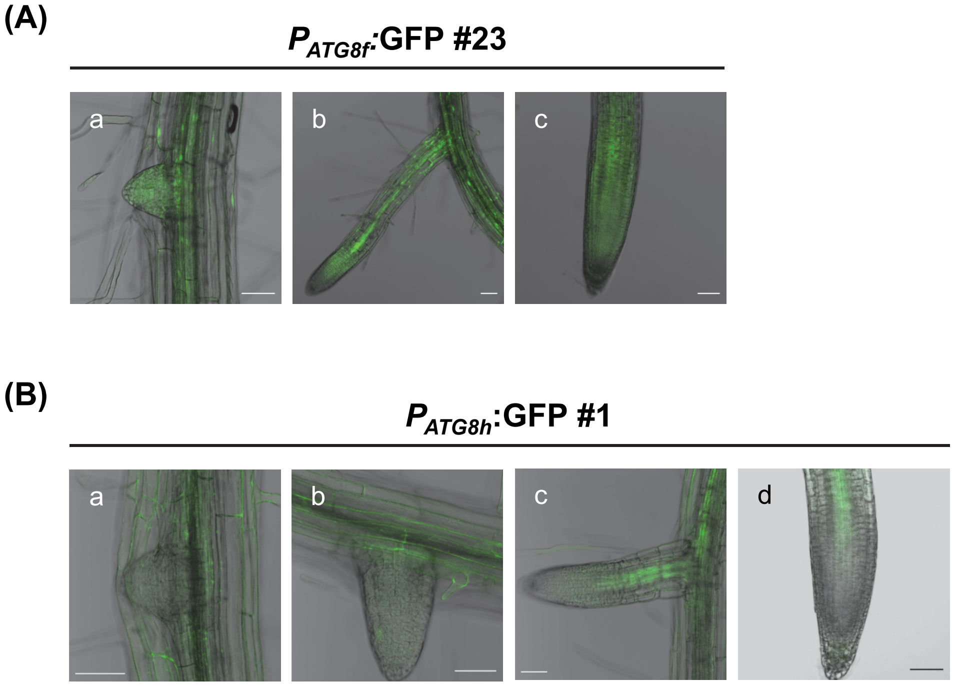

Supplemental Fig. 9 Expression patterns of *At*ATG8f and *At*ATG8h in the lateral root of *Arabidopsis* GFP reporter lines

(A) GFP expression in 9-day-old seedlings of P_{ATG8f}:GFP under Pi-sufficient (a; +P, 250 µM KH_2PO_4) and Pi-deficient (b, c; –P, 0 µM KH_2PO_4, 5 days of starvation) conditions. GFP signals in the lateral root primordia (a), the vascular tissue (b) and the lateral root tip (c). Scale bars = 50 µm.

(B) GFP expression in 9-day-old seedlings of P_{ATG8h}:GFP under Pi-sufficient (a–c; +P, 250 µM KH_2PO_4) and Pi-deficient (d; –P, 0 µM KH_2PO_4, 5 days of starvation) conditions. GFP signals in the lateral root primordia (a, b), the vascular tissue (c) and the lateral root tip (d). Scale bars = 50 µm.

Table S1. The predicted P1BS element and *At*PHR1 binding matrix in the promoter region of *AtATG8f* and *AtATG8h*

**Matrix ID** / **Information**	TF_motif_seq_0434	TFmatrixID_0351
**Sequence LOGO**	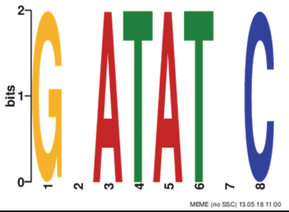	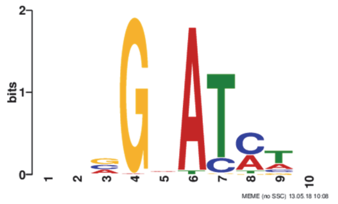
**Description**	PHR1-binding sequence (5′-GNATATNC-3′) found in the upstream regions of phosphate-starvation responsive genes from several plant species	Binding matrix of AtPHR1 (AT4G28610) characterized by protein binding microarray
**Reference**	The prediction of transcription factor binding sites is referred to the Plant Promoter Analysis Navigator (PlantPAN; http://PlantPAN.itps.ncku.edu.tw).	

Table S2. The T-DNA insertion lines for *AtATG8f* and *AtATG8h*

(K.O: knockout; K.D: knockdown)

AGI No.	Gene	Line ID	RT-PCR result
At4g16520	*atg8f-2*	SALK_052510C	K.O
	atg8f-3	SALK_039231	not K.O
	atg8f-5	SALK_133008	K.O
	atg8f-6	SALK_004370	not K.O
At3g06420	*atg8h-2*	SALK_021495	K.D
	atg8h-3	SALK_136493	K.O

Table S4. List of primer sequences used for gene cloning

Gene/AGI No.	Primer name	Sequence (5' to 3')
Promoter constructs		
ATG8f / At4g16520	ATG8f_PacI_pro_exon.for	ttaattaaAGAGACATCAGAACAGCTAACAT
	ATG8f_AscI_SpeI_noSTOP.rev	actagtggcgcgCCTATCAGGATACTTCTCTCTAA
ATG8h/ At3g06420	ATG8h_PacI-2_pro_exon.for	ttaattAAGGTATGAAAGCAGACACAA
	ATG8h_AscI_SpeI_noSTOP.rev	actagtggcgcgcCTATCTGGATATTTCGCAATGATG
Transient dual-luciferase assay reporter constructs		
ATG8f / At4g16520	ATG8f_bucI.rev	actagtCCTTCCACGCCTACACAC
	ATG8f_SacI.for	gagctcTCTCGGTTTTGACATTAATCACTCA
ATG8h/ At3g06420	SacI_ATG8h_pro_exon.for2	gagctcAAGGTATGAAAGCAGACACAAAGAC
	ATG8h_XbaI.rev2	tctagaTACAGATTTCACGACGAATGATTCC
IPS1/ At3g09922	SalI_IPS1_pro.for	ttgtcgacTGAGTGTGTATATGTCTGTG
	IPS1 pro_SpeI.rev	ttactagtATTCTGGAAAGAAGAAACAC
Transient dual-luciferase assay effector constructs		
PHR1 / At1g12370	PHR1_AscI.for	ggcgcgccaATGGAGGCTCGTCCAGTTCATAG
	PHR1_STOPSalI.rev	gtcgacTCAATTATCGATTTTGGGACGCTTTGG
ATAF2/ At5g08790	PacI_ATAF2CDS.for	ttaattaacATGAAGTCGGAGCTAAATTTACCAGCTG
	ATAF2CDS_XhoI.rev	ctcgagTTACCCCTGTGGAGCAAAACTCCAATTC

Table S5. List of primer sequences used for PCR genotyping, RT-PCR and qRT-PCR analyses

Gene/AGI No	Primer name	Sequence (5' to 3')
Genotyping		
ATG8f/ At4g16520	8F-2f	ATGGCAAAAAGCTCGTTCAAGC
	8F-2r	actagtTTATGGAGATCCAAATCCAAATGTG
ATG8h/At3g06420	8H-1f	ggcgcgccaATGGGGATTGTTGTCAAGTCTT
	8H-2r.	actagtTTAGCCGAAAGTTTTCTCGGT
atg8f-2	LB	TGATAGACGGTTTTTCGCCCT
	8F-2r	actagtTTATGGAGATCCAAATCCAAATGTG
atg8f-3	LB	TGATAGACGGTTTTTCGCCCT
	8F-2r	actagtTTATGGAGATCCAAATCCAAATGTG
atg8f-5	8F-1f	ggcgcgccaATGGCAAAAAGCTCGTTCAAG
	LB	TGATAGACGGTTTTTCGCCCT
atg8f-6	LB	TGATAGACGGTTTTTCGCCCT
	8F-2r	actagtTTATGGAGATCCAAATCCAAATGTG
atg8h-2	8H-1f	ggcgcgccaATGGGGATTGTTGTCAAGTCTT
	LB	TGATAGACGGTTTTTCGCCCT
	RB	GGATACTTTCTCGGCAGGAGC
	8F-2r	actagtTTATGGAGATCCAAATCCAAATGTG
atg8h-3	8H-1f	ggcgcgccaATGGGGATTGTTGTCAAGTCTT
	LB	TGATAGACGGTTTTTCGCCCT
	LB	TGATAGACGGTTTTTCGCCCT
	8H-2r	actagtTTAGCCGAAAGTTTTCTCGGT
RT-PCR		
ATG8f/ At4g16520	8F-2f	ATGGCAAAAAGCTCGTTCAAGC
	8F-1r	CTGCTGGAGGAAGAACATTGTCC
ATG8h/ At3g06420	8H-1f	ggcgcgccaATGGGGATTGTTGTCAAGTCTT
	8H-1r	AAGAACCCGTCTTCTTCCTTG
	8H-1f	ggcgcgccaATGGGGATTGTTGTCAAGTCTT
	8H-2r	actagtTTAGCCGAAAGTTTTCTCGGT
ACTIN2/ At3g18780	ACTIN2 For.	TCCAAGCTGTTCTCTCCTT
	ACTIN2 Rev.	GAGGGCTGGAACAAGACTT
qRT-PCR		
ACT8/ At1g49240	qAtACT8.for	CCCAAAAGCCAACAGAGAGA
	qAtACT8.rev	CATCACCAGAGTCCAACACAAT

ATG8a/ At4g21980	qAtATG8a.for1	ACACAAAGATGAGGATGGGTT
	qAtATG8a.rev1	AAGCAACGGTAAGAGATCCAA
ATG8b/At4g04620	qAtATG8b.for1	CCAACTGCGGCATTGATGT
	qAtATG8b.rev1	CCACCAAATGTGTTCTCTCCA
ATG8c/At1g62040	qAtATG8c.for1	TGAGTGCCGAAAAGGCTATC
	qAtATG8c.rev1	ACCAAACCAAAGGTGTTCTCT
ATG8d/At2g05630	qAtATG8d.for2	CCCGAGAAAGCCATCTTCAT
	qAtATG8d.rev2	CGTGTTCTCACCACTGTAACT
ATG8e/At2g45170	qAtATG8e.for2	GTACCTTGTGCCATCAGACC
	qAtATG8e.rev2	AGGAAGCCATCTTCGTCTTT
ATG8f/At4g16520	qAtATG8f.for1	TGGGGCAGTTTGTGTATG
	qAtATG8f.rev1	GGAACCCATCATCATCCTTTT
ATG8g/At3g60640	qAtATG8g.for1	TGTGATTCGTAAGAGAATCCAAC
	qAtATG8g.rev1	CCAAAAGTGTTTTCCCCACT
ATG8h/At3g06420	qAtATG8h.for1	CCAAAGCTCTCTTTGTTTTCG
	qAtATG8h.rev1	AAGAACCCGTCTTCTTCCTTG
	qAtATG8h.for2	TCTTCCTCTGCTCTCTGCAAAC
	qAtATG8h.rev2	CTGGATATTTCGCAATGATGTTGT
ATG8i/At3g15580	qAtATG8i.for1	TGTCAACAACACTCTCCCTCA
	qAtATG8i.rev1	AACCAAAGGTTTTCTCACTGC
GFP	qGFP.for	AGGACGACGGGAACTACAAG
	qGFP.rev	TAAGCTCGATCCTGTTGACG
